# Multifunctional MOF@COF Nanoparticles Mediated Perovskite Films Management Toward Sustainable Perovskite Solar Cells

**DOI:** 10.1007/s40820-024-01390-9

**Published:** 2024-04-11

**Authors:** Yayu Dong, Jian Zhang, Hongyu Zhang, Wei Wang, Boyuan Hu, Debin Xia, Kaifeng Lin, Lin Geng, Yulin Yang

**Affiliations:** 1 MIIT Key Laboratory of Critical Materials Technology for New Energy Conversion and Storage, School of Chemistry and Chemical Engineering, Harbin Institute of Technology, Harbin, 150001 Heilongjiang People’s Republic of China; 2 School of Materials Science and Engineering, East China Jiaotong University, Nanchang, 330013 Jiangxi People’s Republic of China; 3 School of Materials Science and Engineering, Harbin Institute of Technology, Harbin, 150001 Heilongjiang People’s Republic of China

**Keywords:** Perovskite solar cells, Covalent organic frameworks, Metal–organic frameworks, Lead leakage, Stability

## Abstract

Although covalent organic frameworks (COFs) with high *π*-conjugation have recently exhibited great prospects in perovskite solar cells (PSCs), their further application in PSCs is still hindered by face-to-face stacking and aggregation issues. Herein, metal–organic framework (MOF-808) is selected as an ideal platform for the in situ homogeneous growth of a COF to construct a core–shell MOF@COF nanoparticle, which could effectively inhibit COF stacking and aggregation. The synergistic intrinsic mechanisms induced by the MOF@COF nanoparticles for reinforcing intrinsic stability and mitigating lead leakage in PSCs have been explored. The complementary utilization of *π*-conjugated skeletons and nanopores could optimize the crystallization of large-grained perovskite films and eliminate defects. The resulting PSCs achieve an impressive power conversion efficiency of 23.61% with superior open circuit voltage (1.20 V) and maintained approximately 90% of the original power conversion efficiency after 2000 h (30–50% RH and 25–30 °C). Benefiting from the synergistic effects of the in situ chemical fixation and adsorption abilities of the MOF@COF nanoparticles, the amount of lead leakage from unpackaged PSCs soaked in water (< 5 ppm) satisfies the laboratory assessment required for the Resource Conservation and Recovery Act Regulation.

**Supplementary Information:**

The online version contains supplementary material available at 10.1007/s40820-024-01390-9.

## Highlights

- Covalent organic frameworks (COFs) were in situ homogeneously growing on the surface of metal–organic framework (MOF-808) by a covalent bond to construct a core–shell MOF@COF nanoparticle.
- MOF@COF optimized the crystallinity of perovskite to achieve 23.61% efficiency with superior open circuit voltage (1.20 V).
- MOF-assisted COFs to ‘trap’ leaked lead ions by in situ chemical fixation and adsorption.
- The amount of lead leakage (<5 ppm) satisfied the laboratory assessment.

**Supplementary Information:**

The online version contains supplementary material available at 10.1007/s40820-024-01390-9.

## Graphical abstract

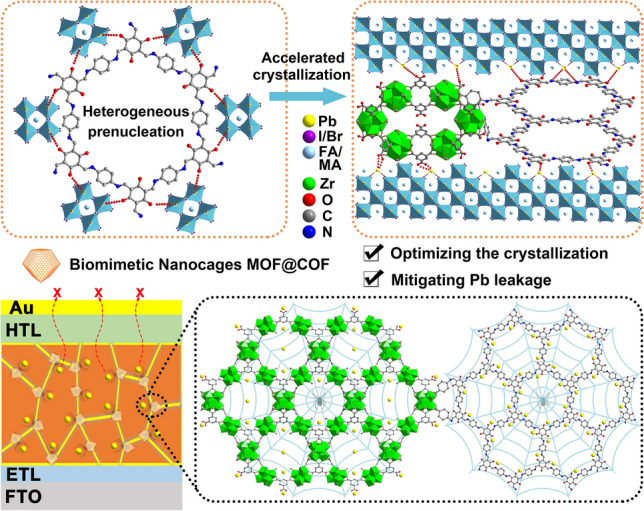

## Introduction

Halide-lead perovskite solar cells (PSCs) have proven to be the most promising photovoltaic technology [1, 2]. The certified PSC power conversion efficiency (PCE) of 26.1% can rival that of market-dominating polycrystalline silicon and is no longer a limiting factor for commercialization [3]. Recently, significant efforts have gradually shifted from pursuing a higher PCE to achieving operational stability and safety [4–6]. The unsatisfactory intrinsic stability of perovskite films mainly stems from the poor crystallization quality and defects produced on the film surface or grain boundaries, which result in unbalanced charge transfer and become charge recombination centers that induce perovskite degradation [7–9]. Once this occurs, heavy metal lead inevitably leaks, threatening the ecological environment and offsetting the competitive advantage of perovskite technology for carbon reduction [6, 10–14]. From this aspect, effectively improving the charge transfer performance and film quality through the use of functionalized materials is of great significance in addressing stability and lead leakage issues to achieve the green and sustainable development of PSCs.

As a novel type of functional organic semiconducting material, covalent organic frameworks (COFs) have recently exhibited great application prospects in perovskite photovoltaic community due to their strong *π*–*π* conjugated skeletons, excellent charge separation efficiency, and multiple functional groups [15, 16]. Among the limited studies [17–24], Zhao’s group pioneered the application of 3D COFs based on spirobifluorene to enhance the performance of PSCs via perovskite—SP-3D COF interaction [17]. These pioneering studies demonstrated that COFs play a crucial role in optimizing the interface and inducing perovskite crystals. However, the face-to-face stacking and aggregation of the COF are major constraints for the further application of COFs in PSCs. From this perspective, the in situ covalent growth of COFs on the surface of a specific platform is a valuable method for further improving their dispersion and charge separation efficiency. Metal–organic frameworks (MOFs) have demonstrated the potential to enhance the performance of PSCs and prevent lead leakage [25–31]. In particular, the abundant formic acids coordinated with the Zr6 cluster could act as an active site of post-synthetic modification, making MOF-808 an ideal platform for the in situ growth of COFs. Meanwhile, MOF-808 exhibits excellent affinity for heavy metal ions due to its porous structure, which could assist COFs to efficiently ‘trap’ leaked lead ions. As a proof-of-concept, the in situ epitaxial growth of COF on MOF-808 will take full advantage of the nanopores and *π*-conjugated skeletons, which endows this hybrid material with more efficient charge transport and capture capacities of leaked lead ions. From the above, we deduce that the well-designed MOF@COF will probably be promising candidates to prepare stable and eco-friendly PSCs.

Herein, TpPa-1-COF was in situ homogeneous growth on the surface of MOF-808 to construct a core–shell MOF@COF nanoparticles, which was applied to address the challenging intrinsic stability of perovskite films and lead leakage issues for the first time. The covalent integration in MOF@COF could efficiently promote the separation and transfer of photogenerated charges. The ordered construction and abundant active sites (C–N, –COO–, and C=O) play a vital role in optimizing the crystallization of large-grained perovskite films and eliminating deep-level defects. MOF@COF nanoparticles mediated perovskite film management endows PSCs with an impressive PCE of 23.61% accompanied by improved stability. More importantly, the large specific surface area and aperture of MOF@COF could effectively chemically chelate Pb ions within perovskite layers and quickly capture leaked Pb^2+^ once PSCs degrade to address the potential environmental risks.

## Experiment

### Synthesis of MOF@COF

#### Synthesis of MOF-808

The ZrOCl_2_·8H_2_O (0.32 g, 1 mmol) dissolved in 20 mL of dimethylacetamide (DMF), then CH_2_O_2_ (20 mL) was added. 1,3,5-benzenetricarboxylicacid (0.21 g, 1 mmol) was added and stirred in an oil bath at 120 °C for 10 h, then the product MOF-808 was separated by centrifugation. The dichloromethane and n-hexane were used to wash production 3 times, then dried in an oven at 100 °C for 6 h to obtain MOF-808.

#### Synthesis of Amino Functionalized MOF-808

MOF-808 (0.2 g) was ultrasonically dispersed in 10 mL of DMF, then p-aminobenzoic acid (0.72 g, 5.2 mmol) was added and reacted at 60 °C for 48 h. The obtained product was obtained after centrifugation and washed with water and ethanol 3 times. Then the product was dried at 100 °C for 12 h to obtain amino-functionalized MOF-808 (NH_2_-MOF-808).

#### Synthesis of MOF-808@TpPa-1-COF

NH_2_-MOF-808 (45 mg) was added to the vacuum tank containing 3 mL DMF and ultrasonic dispersion for 30 min. Then 1,3,5-triformylphloroglucinol (48 mg) and p-Phenylenediamine (32 mg) were added to the mixture and sonicated for 30 min. After sonication is completed, acetic acid (0.5 mL, 3 M) is added in sequence, and the mixture is uniformly mixed and subjected to three freeze–thaw degassing cycles. Then the mixture was heated at 120 °C to react for 72 h, then cool to room temperature and filter to obtain the product and wash the product with tetrahydrofuran. The product was soaked in acetone for 72 h, then dried at 120 °C under vacuum for 12 h to obtain MOF-808@TpPa-1-COF (MOF@COF).

### Device Fabrication

#### Preparation of Substrate and ETL

Perovskite solar cells were prepared with planar structure of FTO/C-TiO_2_/PC_61_BM/Perovskite/HTM/Au. FTO glass (NSG Company, 15 Ω m^−2^ resistance) was etched by 3 M hydrochloric acid and zinc powder, and washed in successive with detergent, deionized water, acetone, and isopropanol in an ultrasonic bath for 15 min, respectively. The dry glass was treated with ozone for 20 min. The blocking titanium dioxide layer was prepared by mixing titanium (IV) isopropanol (175 μL) and hydrochloric acid (17.5 μL 3 mol L^−1^) in 1.25 mL isopropanol and spin-coating at 3000 rpm for 30 s. Then the substrate was sintered at 500 °C for 30 min. After cooling, PCBM with 5 mg mL^−1^ was spin-coated on ETL at 3000 rpm for 30 s, and heated at 70 °C for 10 min.

#### Preparation of Perovskite Precursor Solution

The triple cation perovskites of the generic form (FAPbI_3_)_0.93_(MAPbBr_3_)_0.04_(CsPbI_3_)_0.03_ was prepared by mixing 1.53 M PbI_2_, 1.4 M FAI, 0.5 M MACl, and 0.0122 M MAPbBr_3_ into 1 mL DMF/ DMSO (dimethyl sulfoxide) (8:1 v/v). Then, perovskite precursor solution was stirred for 12 h to ample dissolution and filtrated using a 0.45 μm syringe filter before spinning. Perovskite solutions were successively spin-coated on the ETL substrates at 1000 and 5000 rpm for 10 and 30 s, respectively. During the last 15 s, 150 μL of chlorobenzene was poured onto spinning substrate. Especially, different concentration of MOF@COF was added to chlorobenzene to obtain functionalized perovskite films. Afterward, the films were annealed at 120 °C for 1 h in a nitrogen filled glove box. Then oFPEAI (20 mM in isopropanol) was spin-coated at 4000 rpm for 30 s and annealed at 100 °C for 5 min. HTM solution was prepared by dissolving 72.3 mg Spiro-OMeTAD in 1 mL CB and doped with 29 μL 4-tert-butylpyride (TBP), 17.5 μL bis(trifluoromethylsulfonyl)imide lithium salt (Li-TFSI) solution (520 mg in 1 mL acetonitrile), 30 μL FK209 solution (300 mg in 1 mL CN) which was spin-coated on perovskite film at 4000 rpm for 20 s. Finally, about 80 nm of Au electrode was thermally evaporated on the HTL film.

## Results and Discussion

### Characterization of MOF@COF

**Fig. 1:**
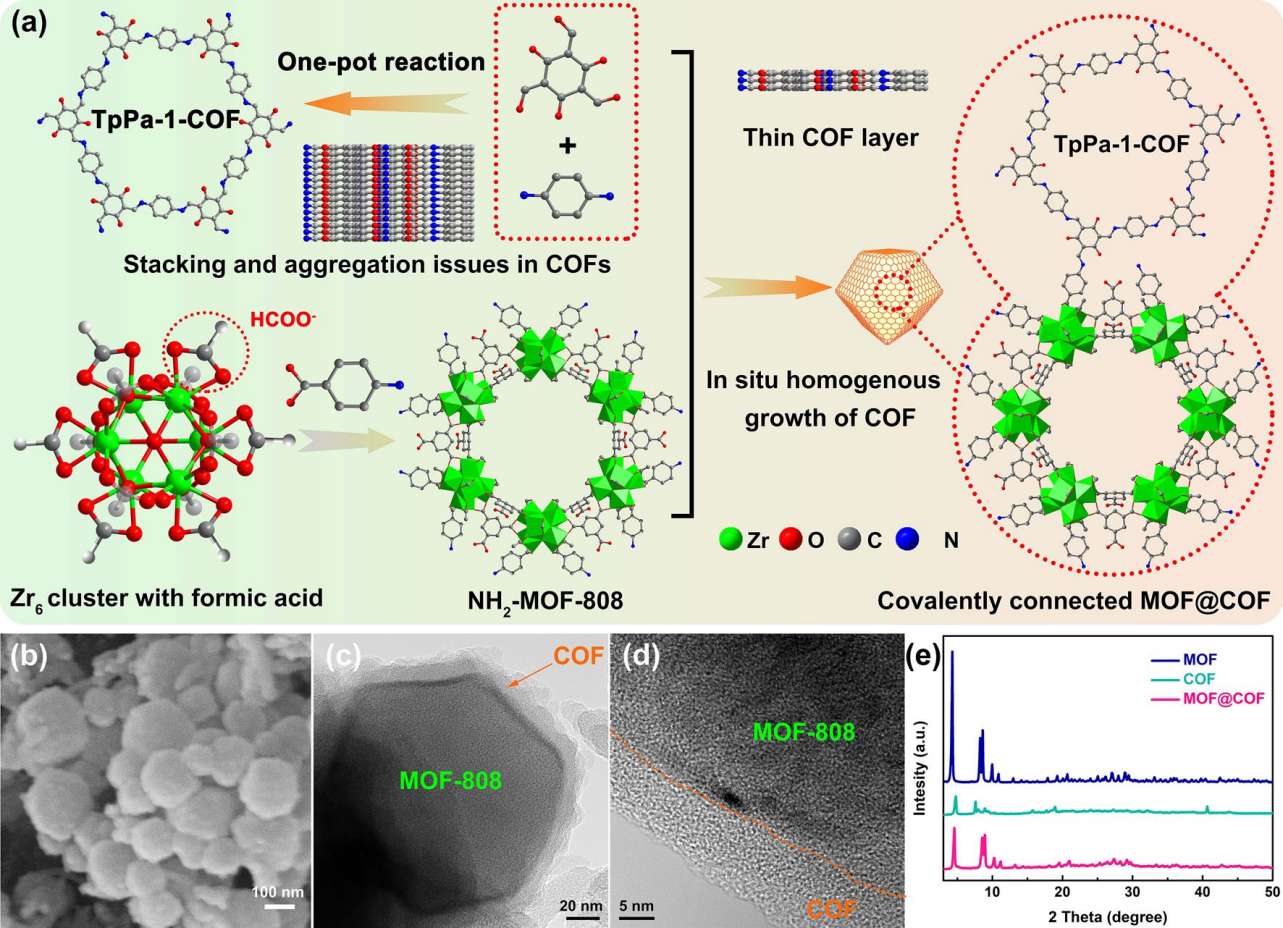
**a** Preparation process of the MOF@COF. Scanning electron microscopy (SEM) images of **b** MOF@COF. **c** Transmission electron microscopy (TEM) and **d** high-resolution (HR)-TEM images of MOF@COF. **e** XRD patterns

As illustrated in Fig. 1a, the stirring method was used to prepare MOF-808 nanocrystals with a small size of approximately 200 nm (Fig. S1) by regulating the reaction time [32]. Subsequently, a large excess of *p*-aminobenzoic acid reacted with MOF-808 to guarantee enough –NH_2_ groups in the surface of MOF-808 (NH_2_-MOF-808), which substituted for the original formic acid coordinated with the Zr6 cluster to generate a ‘link bridge’ for covalently reacting during the subsequent reaction with TpPa-1-COF. This modification strategy did not significantly change the morphology of MOF-808. For post-synthetic modification, NH_2_-MOF-808 was introduced into the facile in situ reaction of TpPa-1-COF with 1,3,5-trimethoxy-phloroglucinol and p-phenylenediamine in *N*,*N*-dimethylformamide to synthesize MOF@COF core–shell nanostructures. As shown in Fig. 1b, the core–shell MOF@COF nanoparticles exhibit a rougher surface morphology after being uniformly covered with TpPa-1-COF. High-resolution transmission electron microscopy (HR-TEM) (Fig. 1c, d) revealed that the MOF-808 polyhedral nanostructure acted as the core, and TpPa-1-COF with a thickness of 20 nm served as the shell layer coating the NH_2_-MOF-808 surface. This in situ epitaxial growth strategy can effectively inhibit face-to-face stacking and aggregation of the COF. High-angle annular dark-field scanning transmission electron microscopy (HAADF-STEM) and elemental mapping images confirmed that Zr was uniformly distributed throughout the nanostructure (Fig. S1c). Additionally, powder X-ray diffraction (PXRD), X-ray photoelectron spectroscopy (XPS), and Fourier-transform infrared (FT-IR) spectroscopy were conducted to further confirm the MOF@COF nanostructure. The PXRD patterns (Fig. 1e) and XPS spectra (Fig. S2a) of the MOF@COF contain the characteristic peaks of MOF-808 and TpPa-1-COF. The typical peaks of MOF-808 and TpPa-1-COF at 1378 cm^−1^ (–COOH) and 1251 cm^−1^ (C–N) appeared in the FT-IR spectrum of the MOF@COF (Fig. S2b). These results confirm the successful preparation of core–shell MOF@COF nanoparticles. Compared to MOF-808, the UV–Vis diffuse reflectance spectrum (DRS) of the MOF@COF showed a one-reflection band edge similar to that of TpPa-1-COF, which confirmed the formation of covalent bonds between TpPa-1-COF and NH_2_-MOF-808 through the Schiff base reaction (Fig. S3a). The UV–Vis absorption spectra showed that MOF-808 exhibited intense absorption in the UV region (200–300 nm). After the introduction of TpPa-1-COF, the absorption region of the MOF@COF expanded to 600 nm, which was ascribed to the excellent light-absorption properties of TpPa-1-COF (Fig. S3b). Therefore, the optical properties endow MOF@COF with excellent UV-filtering capability to filter high-energy photons, which will enhance the light utilization ratio to achieve higher photocurrent, performance of PSCs through down conversion.

### Crystallization Optimization of Perovskite Films Induced by MOF@COF

**Fig. 2:**
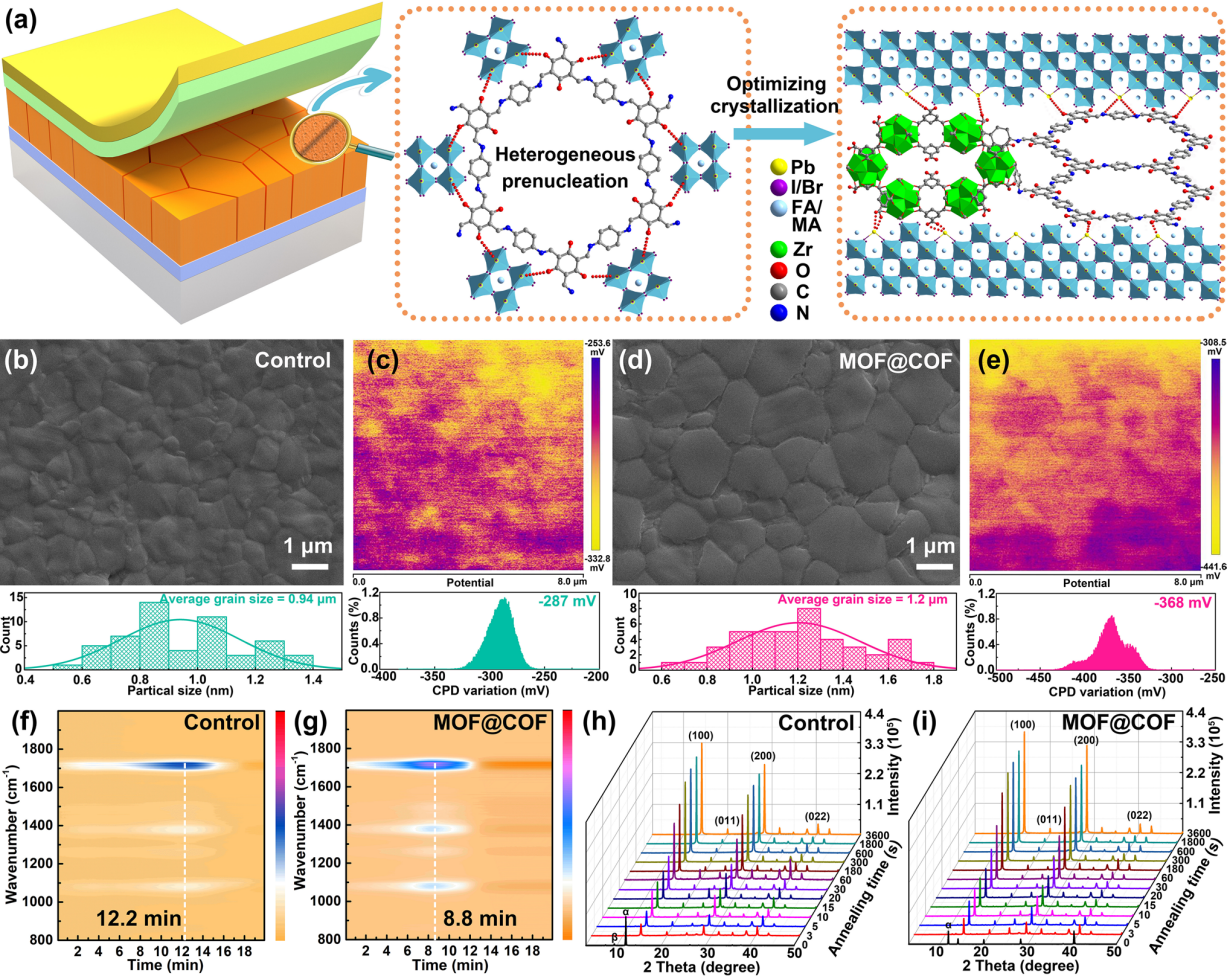
**a** Schematic diagram of MOF@COF-functionalized perovskite films. SEM and KPFM images for **b**, **d** control and **c**, **e** MOF@COF-functionalized perovskite films. In situ TG-FTIR spectra of perovskite precursor solution **f** as a control and **g** with the MOF@COF. In situ XRD measurement of **h** control and **i** MOF@COF-functionalized perovskite films

Based on its unique structural features, the MOF@COF was incorporated into a perovskite precursor solution to obtain high-quality eco-friendly PSCs. As shown in Fig. 2a, the MOF@COF can act as a heterogeneous prenucleation center to precisely regulate and optimize the crystallization of large-grained perovskite films and eliminate defects through strong chemical interaction between Pb^2+^ and I^−^ with the C–N, –COO^−^, and C=O active groups. In addition, the structural features of the MOF@COF nanostructure (highly ordered and intrinsic pores) induced the growth of highly oriented perovskite films [20]. Notably, the introduction of a heterogeneous prenucleation center reduced the critical free energy of crystal nucleus formation, which further accelerated the nucleation process [33, 34]. To achieve the optimal doping effect, different MOF@COF concentrations (mg mL^−1^) were introduced into the perovskite precursor solution. At 1.5 mg mL^−1^, the PSCs exhibited the optimal photovoltaic performance, and this concentration was applied in the following experiments (Fig. S4 and Table S1).

Nucleation and growth have a strong impact on the crystallization growth of large-grained perovskite films [35, 36]. As illustrated in Figs. S5 and S6, TEM and SEM mapping confirmed that the characteristic elements of the MOF@COF were evenly distributed in the perovskite films. Meanwhile, the amorphous area was distinguished in the contiguous perovskite grains from the HRTEM images (Fig. S5b), indicating that the gathering of MOF@COF nanoparticles is similar to that in previous studies [37–39]. From the fast Fourier transform (FFT) photographs and lattice distances, the calculated plane distance of 3.0 Å matched well with the (100) reflection of the perovskite phase. This well-ordered nanomaterial acted as a heterogeneous prenucleation center to precisely optimize the crystallization of large-grained perovskite films. As displayed in SEM (Fig. 2b, d) and atomic force microscopy (AFM) images (Fig. S7), the average grain size and root-mean-square roughness of MOF@COF-functionalized perovskite films were 1.2 and 29.6 nm, respectively, which are larger than those of control films (0.94 and 28.6 nm). This was mainly ascribed to the formation of a highly oriented perovskite film induced by the MOF@COF. In addition, the effect of the MOF@COF on the surface potential of the perovskite films was investigated using Kelvin probe force microscopy (KPFM) [2]. As shown in Fig. 2c, e, the electronic chemical potential distribution for the MOF@COF-functionalized perovskite film was − 368 mV, which was larger than that of the control film (− 287 mV). The strong chemical interaction between the perovskite and MOF@COF and the elimination of defects induced a uniform distribution of the surface potential, which was beneficial for efficient charge transfer to inhibit nonradiative recombination.

To understand the effect of the MOF@COF on the precise regulation of the crystallization process of perovskite films, a series of in situ experiments were conducted. During the in situ thermogravimetric-FTIR (TG-FTIR) analysis depicted in Fig. 2f, g, the perovskite precursor solution was heated at 100 °C for 1 h under a N_2_ atmosphere to in situ monitor the evaporation times of organic solvents [40]. For the control solution, the maximum volatilization rate of organic solvents occurred at 12.2 min, which was significantly shortened to 8.8 min after introducing the MOF@COF. The selected FT-IR spectra at maximum volatilization time (Fig. S8) shows that the peaks located at 1711 and 1081 cm^−1^ are assigned to the stretching vibration of C=O in DMF and S=O in DMSO, respectively. Therefore, the organic solvents (DMF and DMSO) in the perovskite precursor solution evaporated quickly from the intermediate phase after integration with the MOF@COF, and the shortened nucleation process promoted the formation of highly crystalline perovskite films. During the annealing process, in situ XRD measurements were performed at different annealing times to monitor the phase transition process (Fig. 2h, i) [41]. Initially (0 s), the control film showed more substantial diffraction peaks of PbI_2_ (α) and nonperovskite phase (β) compared with the MOF@COF-functionalized perovskite film. After thermal annealing, both films exhibited rapid phase conversion. Simultaneously, the diffraction peak intensity of the perovskite phase (14.1°) in the XRD pattern of the MOF@COF-functionalized perovskite film was stronger than that of the control film. This implies that the MOF@COF can efficiently accelerate solvent evaporation to promote the conversion of the intermediate phase to the perovskite phase and agrees with the TG-FTIR results for the MOF@COF-functionalized perovskite film.

### Interaction Between MOF@COF and Perovskite

**Fig. 3:**
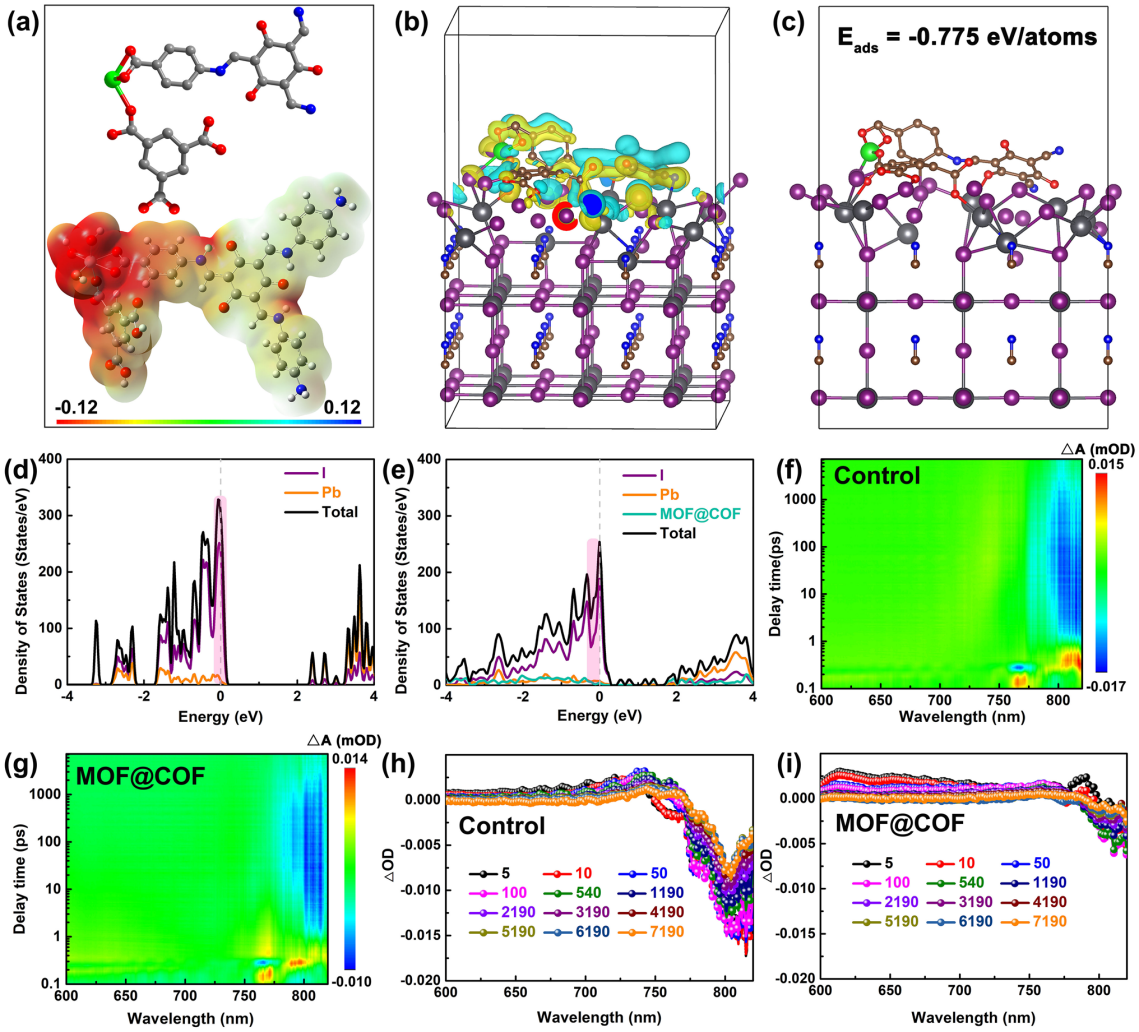
**a** Selected fragment and ESP in the MOF@COF. **b** Theoretical models of perovskite with the MOF@COF. **c** DFT charge difference calculations. DOS **d** before and **e** after the introduction of MOF@COF-passivated perovskite. 2D pseudocolor images and TAS spectra for **f**, **h** control and **g**, **i** MOF@COF

Additionally, density functional theory (DFT) calculations were used to investigate the effect of the MOF@COF on the crystal defects [42, 43]. The selected fragment that best represents the MOF@COF, and its electrostatic potential (ESP) is shown in Fig. 3a. The highest electron density (red) in the MOF@COF was attributed to C=O (carboxylate groups), –OH (hydroxide radical), and C–N. Based on the optimized structure, the differential charge density distribution is illustrated in Fig. 3b, where blue and yellow represent electron depletion and accumulation, respectively [44]. To gain further insight into the alteration of the formation energies of the Pb and I defects, the interactions between the MOF@COF and the perovskite are displayed in Fig. 3c. These electron-rich active groups could act as Lewis acids and bases to anchor the uncoordinated Pb ions in a crystal lattice in situ or hydrogen bond with ammonium cations to reduce the formation of deep traps and inhibit nonradiative recombination. This enabled enhanced absorption of the MOF@COF onto the Pb and I defect with a strong adsorption energy of − 0.775 eV atom^−1^. The density of states (DOS) (Fig. 3d, e) further confirmed the elimination of trap states [44]. After introducing the MOF@COF, the DOS of Pb, I, and total defects decreased compared to those of the control perovskite film, indicating that MOF@COFs with electron-rich active groups could efficiently eliminate the defect state density to achieve high-performance PSCs.

Subsequently, space-charge-limited current (SCLC) measurements with the electron-only structure of FTO/TiO_2_/perovskite/PCBM/Ag were conducted to demonstrate defect elimination [45, 46]. From Fig. S9, the trap-filled limited voltage (*V*_TFL_) of the control device was calculated to be 0.80 V, which was much larger than that of the MOF@COF-functionalized device (0.59 V). According to the equation: *V*_TFL_ = *N*_t_*eL*^2^/2*εε*_0_, the corresponding trap densities (*N*_t_) were 1.50 × 10^16^ and 1.11 × 10^16^ cm^−3^ for devices without or with the MOF@COF. XPS spectra were obtained to further investigate the chemical interactions between the MOF@COF and perovskite films. The binding energies of Pb_4*f*7/2_ and Pb_4*f*5/2_ shifted from 137.3 and 142.2 eV in control perovskite films to 137.9 and 142.8 eV (Fig. S10a), and the I_3*d*5/2_ and I_3*d*3/2_ binding energies also shifted toward higher binding energies (Fig. S10b) for the MOF@COF-functionalized perovskite film. Meanwhile, the O 1*s* and N 1*s* bond energy changes (Fig. S10c, d) also confirmed that the C=O and C–N active groups play a crucial role in eliminating the uncoordinated Pb^2+^ and I^−^ defects. Additionally, femtosecond transient absorption spectroscopy (fs-TAS) was performed to explore the charge transport dynamics [47, 48]. As illustrated in Fig. 3f, g, the 2D pseudocolor spectra demonstrate that the negative ground-state bleaching (GSB) produced by the femtosecond laser excitation was distributed in the range of 760–820 nm, which was assigned to the charge depletion of the valence band. The typical $\Delta$OD versus wavelength at different delay times is shown in Fig. 3h, i. Compared to the control perovskite film, the MOF@COF-functionalized perovskite film exhibited a much weaker negative peak intensity, indicating faster extraction and transfer of photogenerated charges to inhibit nonradiative recombination. Therefore, preparing hysteresis-free high-efficiency PSCs is essential.

Additionally, the steady-state photoluminescence (PL) and time-resolved photoluminescence (TRPL) spectra of different perovskite films on glass were recorded [49]. After incorporating the MOF@COF, the significantly heightened PL intensity indicated efficient defect elimination through active groups (C=O and C–N) bonding with the defect sites (Pb^2+^ and I^−^) in the perovskite films (Fig. S11). Meanwhile, the average TRPL lifetime of the control film was 434 ns, which was prolonged to 699 ns for the MOF@COF-functionalized film (Table S2), further demonstrating efficient charge extraction and transport dynamics.

### Photovoltaic Performance of PSCs

Given the positive effects of the MOF@COF in eliminating defects and regulating the crystallization of perovskite films, the performance of the MOF@COF-functionalized PSCs was investigated using the plain n-i-p structure of FTO/electron transport layer/perovskite/hole transport layer (HTL)/Au. As illustrated in Fig. S12, the cross-sectional SEM image of the PSCs confirmed that the ultra-large perovskite grains were in close contact with the other functional layers, ensuring excellent charge transfer. Ultraviolet photoelectron spectroscopy (UPS) and UV–Vis absorption spectroscopy (Fig. S13) were employed to investigate the electronic configurations of the perovskite films with and without the MOF@COF [50]. The work function (*W*_F_) and the valence band levels with respect to vacuum were calculated to be − 4.44, − 4.61, − 5.55, and − 5.43 eV for perovskite films without and with the MOF@COF (Fig. S13a). Accordingly, the incorporation of the MOF@COF induces an up-shift in the Fermi energy (*E*_F_) level of the perovskite films, leading to a well-matched energy-level alignment between the perovskite films and the Spiro-OMeTAD-based HTL system (Fig. S13b).

**Fig. 4:**
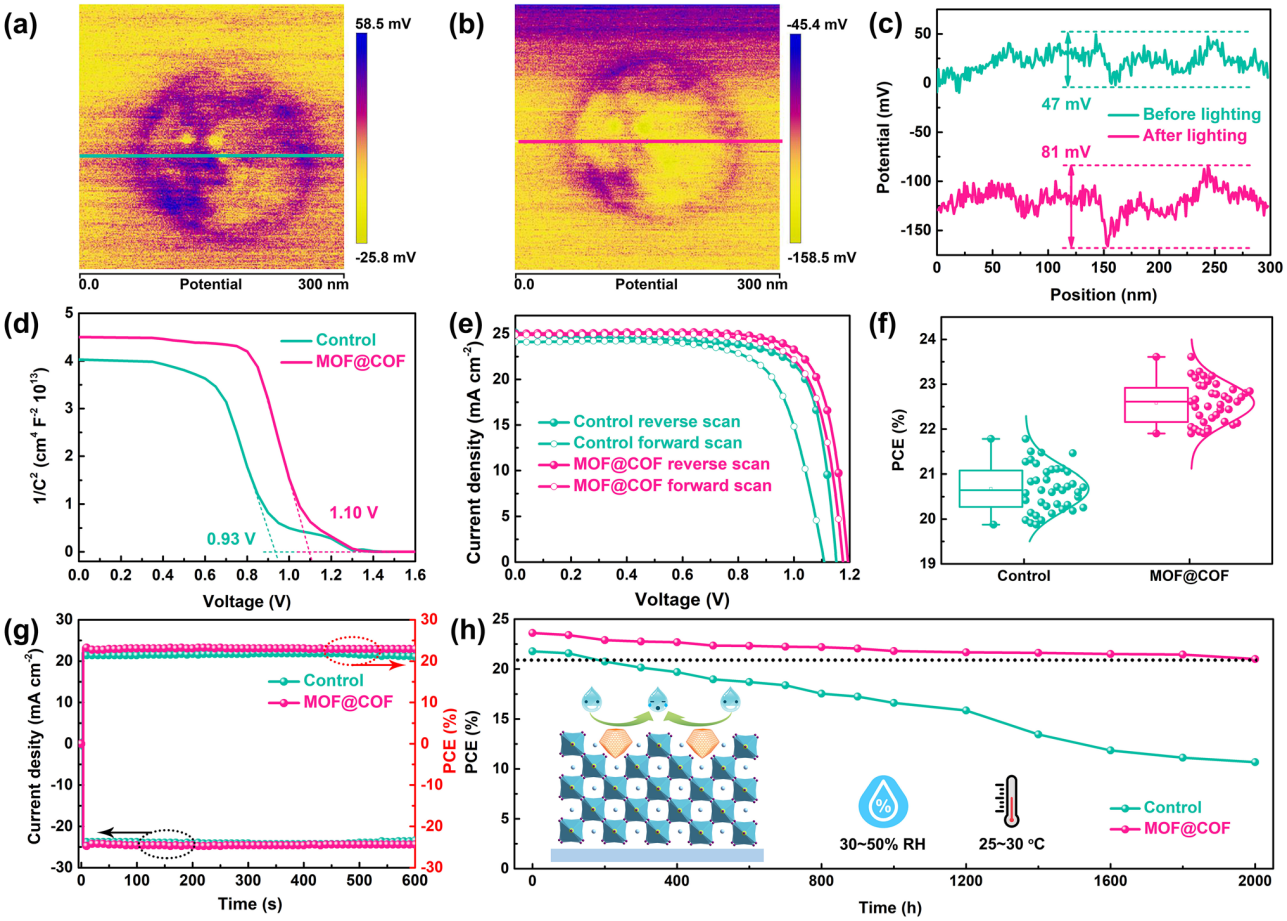
KPFM images of the MOF@COF **a** without irradiation and **b** with irradiation for 10 min. **c** Relevant potential distribution. **d** Mott-Schottky curves. **e** *J–V* curves of different PSCs with different scan directions. **f** Statistical PCEs of 40 PSCs. **g** Stabilized power output at maximum power tracking. **h** Long-term stability

Based on the above analysis, the incorporation of the MOF@COF resulted in high-quality perovskite films with enhanced optical and electrical properties. The surface potential of the MOF@COF nanomaterial before and after irradiation was first investigated using KPFM. As shown in Fig. 4a–c, the surface potential difference of the MOF@COF was approximately 47 mV in the dark and increased to 81 mV under illumination, demonstrating the increased surface charge density. On the one hand, the abundant conjugated system endow TpPa-1-COF with excellent electron transfer capacity to promote efficient seperation and transfer of photogenerated charges. On the other hand, the covalent bond formed between the NH_2_-MOF-808 and TpPa-1-COF serves as a bridge for efficient migration of photogenerated charge in bulk phase. Therefore, the surface potential significantly increased by ≈34 mV under illumination, demonstrating that a built-in electric field will be formed to stimulate the efficient extraction and transfer of photogenerated charges in PSCs. Mott–Schottky curves were obtained to confirm this statement [44, 51]. As shown in Fig. 4d, the built-in potential (*V*_bi_) value for control PSCs was 0.93 V, which was much smaller than that for MOF@COF-functionalized PSCs (1.10 V). Therefore, the incorporation of the MOF@COF provides the driving force for the extraction and transport of photogenerated charges to inhibit defect-induced nonradiative recombination, which contributes to improving the photovoltaic performance of PSCs.

The *J–V* curves in Fig. 4e show that the best PCE of PSCs based on the MOF@COF was 23.61%, coupled with a short-circuit current (*J*_SC_) of 25.18 mA cm^−2^, a fill factor (FF) of 0.78, and a surprised *V*_OC_ of 1.20 V. Notably, this PCE surpasses those of most reported devices based on a single component COFs or MOFs (Table S3). The photovoltaic parameters of the optimal PSC were significantly higher than those of the control PSCs (PCE = 21.78%, *V*_OC_ = 1.17 V, *J*_SC_ = 24.92 mA cm^−2^, and FF = 0.75). Meanwhile, the hysteresis index decreased from 12.9% for the control PSCs to 5.8% for the target PSCs, which was mainly ascribed to defect elimination and the high-quality perovskite film induced by the MOF@COF (Table S4). Meanwhile, the lower current density of the MOF@COF-functionalized PSCs under dark conditions was attributed to efficient charge separation and transfer (Fig. S14a), which was also confirmed by electrical impedance spectroscopy (EIS) measurements (Fig. S14b). Subsequently, 40 different PSCs were collected to evaluate the repeatability of the in situ chemical fixation strategy; the relevant parameter distributions are shown in Figs. 4f and S15. In particular, the average PCE of the control PSCs was 20.65%, which significantly increased to 22.55% after integration with the MOF@COF, and all the statistical parameters of the target PSCs were significantly improved compared with those of the control PSCs. Due to the excellent light response of the MOF@COF, the enhancement of incident photon-to-electron conversion efficiency (IPCE) was consistent with the slightly improved light absorption, leading to an increase in the integrated *J*sc from 23.54 and 24.15 mA cm^−2^, which agrees well with the *J–V* values (Fig. S16).

Moreover, the effect of the MOF@COF on the stability of unencapsulated PSCs was evaluated under various test conditions. As illustrated in Fig. 4g, the MOF@COF-functionalized PSCs delivered a stabilized PCE of 22.91%, which was larger than that of 21.10% for the control PSCs after continuing AM 1.5G illumination for 600 s at the maximum power point (MPP) [52]. In the surrounding environment (30–50% RH and 25–30 °C), the MOF@COF-functionalized PSCs still maintained approximately 90% of the original PCE after 2000 h; however, the control PSCs lost approximately 60% of the PCE (Figs. 4h and S17). The improved stability was ascribed to the stabilization of the perovskite lattice through the in situ chemical fixation effect of the MOF@COF on defect elimination and crystallization regulation in high-quality perovskite films. The intrinsic stability of the MOF@COF can resist the erosion of humidity and protect PSCs stability. The improved stability was further confirmed by the operational stability test under the MPP condition and thermal stability test under thermal annealing at 85 °C in nitrogen condition (Fig. S18).

### Effect of MOF@COF on Inhibiting Lead Leakage

**Fig. 5:**
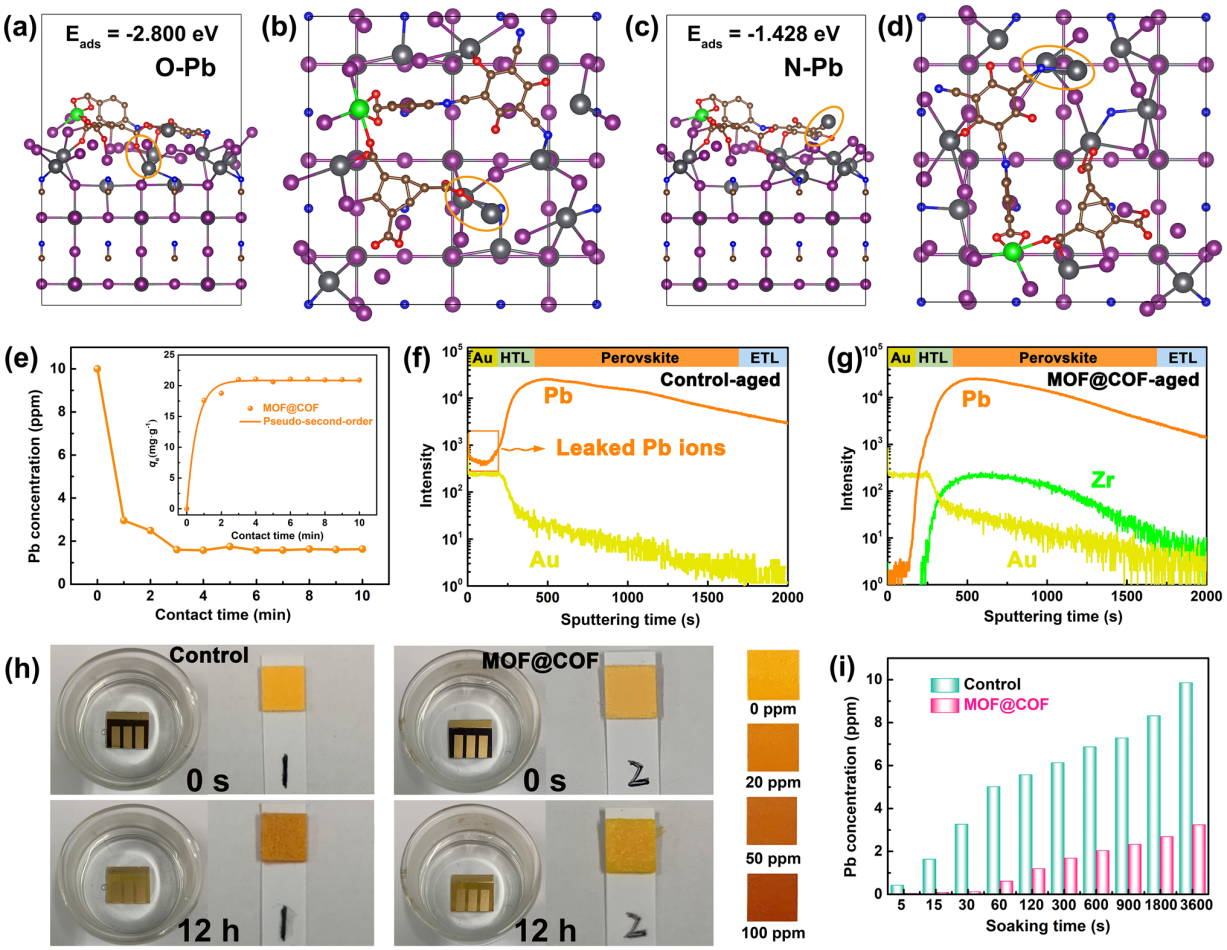
Structure models for O (**a** side view and **b** bird view) and N atoms (**c** side view and **d** bird view) adsorbing leaking Pb^2+^ ions. **e** Pb concentration changes with contact time (Inset: pseudo-second-order model). TOF–SIMS depth profiles for** f** aged control and** g** MOF@COF-functionalized PSCs. Pb concentration in the polluted water was detected by **h** Pb^2+^ testing paper and **i** ICP-OES

The inherently large surface area (Fig. S19) and porous structure endow MOFs and COFs with an excellent adsorption capacity for heavy metal ions, which prompted us to explore the in situ chemical fixation and adsorption of a MOF@COF in mitigating Pb leakage in PSCs [53–58]. DFT calculations were conducted to compare the interaction between the perovskite and N and O atoms in the MOF@COF, as shown in Fig. 5a–d. The adsorption energy *E*_ads_ was calculated according to the following equation: *E*_ads_ = *E*_Pero-MOF@COF-Pb_-*E*_Pero-MOF@COF-_*μ*_Pb_, where* E*_Pero-MOF@COF-Pb_, *E*_Pero-MOF@COF_, and *μ*_Pb_ are the total energy of the adsorption configuration, the total energy of Pero-MOF@COF, and chemical potential of Pb, respectively. Accordingly, the *E*_ads_ values of N and O for leaking Pb^2+^ ions were − 1.428 and − 2.800 eV, demonstrating that the active groups of C–N, –COO^−^, and C=O exhibited a strong affinity for Pb^2+^ ions. The results of the adsorption kinetics and isotherms showed that the maximum Pb adsorption capacity obtained from the pseudo-second-order model was 427.9 mg g^−1^ (Figs. 5e and S20) [53]. These combined results indicate that the MOF@COF can effectively capture Pb^2+^ to reduce the risk of Pb leakage. To further monitor the migration path of the leaked Pb^2+^ ions, the depth profiles of Pb^2+^ ions in the aged PSCs were investigated using time-of-flight secondary ion mass spectrometry (ToF–SIMS) [54]. As illustrated in Fig. 5f, g, a Pb^2+^ signal appeared on the Au electrode, indicating that Pb ions easily migrated into the environment and seriously threatened the ecosystem. Interestingly, only a small trace Pb^2+^ signal was observed on the Au electrode for the MOF@COF-functionalized PSCs, which demonstrated that the MOF@COF could effectively trap Pb ions through the synergistic effect of in situ chemical fixation and adsorption. Finally, the PSCs were immersed in water for different durations to investigate the amount of Pb that leaked from the degraded PSCs. As shown in Fig. 5h, the Pb concentration in the contaminated water was quickly monitored by the color of the Pb^2+^ testing paper for the control PSCs, which was much darker than that of the MOF@COF-functionalized PSCs after 1 h. Inductively coupled plasma optical emission spectroscopy (ICP-OES) was used to precisely determine the Pb concentration. As shown in Fig. 5i, the target PSC exhibits a much lower Pb concentration than the control PSC, thereby reducing the risk of Pb leakage. Based on the above analysis, the MOF@COF played a crucial role in mitigating Pb leakage. The C–N, –COO^−^, and C=O active groups in the MOF@COF could in situ chemically fix uncoordinated Pb^2+^ to strengthen the internal stability of the perovskite film. However, even if the perovskite degrades, the biomimetic nanoparticles of the MOF@COF could also act as an adsorbent (like a spider web) to “trap” Pb^2+^ ions through an in situ chemical adsorption method.

## Conclusions

In summary, core–shell MOF@COF nanoparticles were constructed by the in situ epitaxial growth of a homogenous COF on the surface of a MOF-808 platform, which was applied to address the challenging stability and Pb leakage issues in PSCs for the first time. This design boosts the interface interaction of the MOF with the COF and endows the MOF@COF with a more efficient charge transport ability, which is superior to the common stacked and aggregated structure of COFs. The experimental results and DFT calculations demonstrated that the synergistic effect of the MOF@COF results in strong chemical interactions with the perovskite components, which can optimize the crystallization of large-grained perovskite films with minimal defects. The resultant PSC yielded a significantly improved PCE of 23.61% and *V*_OC_ of 1.20 V, accompanied by substantially enhanced long-term stability. More importantly, the biomimetic nanoparticles MOF@COF create “traps” to strategically address the possible Pb leakage risks through the dual function of in situ chemical fixation and adsorption. This is the first proof-of-concept approach for developing stable and eco-friendly PSCs by taking advantage of TpPa-1-COF and a MOF.

## Supplementary Information

Below is the link to the electronic supplementary material.

Supplementary file1 (PDF 5727 KB)
