# Supplementary material for: Multifunctional MOF@COF Nanoparticles Mediated Perovskite Films Management Toward Sustainable Perovskite Solar Cells
Source: Nanomicro Lett. 2024 Apr 11;16:171. doi: 10.1007/s40820-024-01390-9 (PMC11009200; doi:10.1007/s40820-024-01390-9)
Supplement: Supplementary file 1 — Supplementary file1 (PDF 5727 KB) [file 40820_2024_1390_MOESM1_ESM.pdf]

Supporting Information for

# Multifunctional MOF@COF Nanoparticles Mediated Perovskite Films Management Toward Sustainable Perovskite Solar Cells

Yayu Dong[1, 2], Jian Zhang[1, *], Hongyu Zhang[1], Wei Wang[1], Boyuan Hu[1], Debin Xia[1], Kaifeng Lin[1], Lin Geng[3], and Yulin Yang[1, *]

[1]MIIT Key Laboratory of Critical Materials Technology for New Energy Conversion and Storage, School of Chemistry and Chemical Engineering, Harbin Institute of Technology Harbin 150001, Heilongjiang, P. R. China

[2]School of Materials Science and Engineering, East China Jiaotong University, Nanchang, 330013, Jiangxi, P. R. China

[3]School of Materials Science and Engineering, Harbin Institute of Technology, Harbin 150001, Heilongjiang, P. R. China

*Corresponding authors. E-mail: ylyang@hit.edu.cn (Yulin Yang); zhaji@hit.edu.cn (Jian Zhang)

## S1 Experimental Procedures

***Materials and solvents***: All the reagents were purchased from commercialization without further purification. Solvents for the device fabrication (N,N-Dimethylformamide (DMF), acetonitrile (CN), dimethyl sulfoxide (DMSO), chlorobenzene (CB) and isopropanol (IPA)) were purchased from Sigma-Aldrich. $CsPbI_2$, methylammonium chloride (MACl), 2-(2-Fluorophenyl)ethylamine iodide (oFPEAI), formamidinium iodide (FAI), methylammonium bromide (MABr), 4-tert-butylpyride (TBP), FK209, and bis(trifluoromethylsulfonyl)imide lithium salt (Li-TFSI) were obtained commercially from Xi'an Polymer Light Technology Corp. Lead iodide ($PbI_2$) and lead bromide ($PbBr_2$) were purchased from Alfa. Spiro-OMeTAD was obtained commercially from Toronto Research Chemicals, Inc. Additionally, $ZrOCl_2 \cdot 8H_2O$, 1,3,5-benzenetricarboxylicacid, p-Phenylenediamine were purchased from Aladdin. 1,3,5-triformylphloroglucinol (Tp) synthesized according to the literature method .[1]

***Density functional theory (DFT) calculations***: The first principles calculation was used to calculate the adsorption of perovskite chalcogenide (001) on MOF@COF. Its adsorption energy and the charge density difference of the adsorption configuration have been calculated. At the same time, the adsorption energies of MOF@COF on Pb ions in the post-adsorption conformation were also calculated. This work has employed the Vienna ab initio simulation package (VASP) to perform all density functional theory (DFT) calculations within the generalized gradient approximation (GGA) using the Perdew-Burke-Ernzerhof (PBE) function. the projected augmented wave (PAW) potentials were chosen to describe the ionic cores and take valence electrons into account using a plane wave basis set with a kinetic energy cutoff of 500 eV. Partial occupancies of the Kohn−Sham orbitals were allowed using the Gaussian smearing method and a width of 0.05 eV. The electronic energy was considered self-consistent when the energy change was smaller than $10^{-6}$ eV. A geometry optimization was considered convergent when the force change was smaller than 0.01 eV/Å. Grimme's DFT-D3 methodology was used to describe the dispersion interactions. The Brillouin zone was sampled using Monkhorst-Pack mesh k-points with a reciprocal space resolution of $2\pi \times 0.04/\text{Å}$.

***Characterization***: The photocurrent-voltage (J-V) plots and Electrical impedance spectroscopy (EIS) were performed at the air atmosphere with a Gamry Framework and a solar simulator (Newport) under AM 1.5 G sunlight produced by a 450 W xenon lamp. Active area of PSCs is 0.06 $cm^2$. The incident photo to electron efficiency (IPCE) spectra were conducted under the excitation beam from a 300 W xenon lamp. And a 1/4 monochromator (model 74125 Oriel Cornerstone 260, Newport, USA) was used to collect the given light. Fourier transform infrared (FT-IR) spectra were carried out using a PerkinElmer Spectrum 100 FT-IR spectrometer. Shimadzu XRD-6000 X-ray Diffraction instrument with Cu-Kα radiation was used to conduct X-ray diffraction (XRD) patterns. For in situ XRD test, the perovskite solutions were successively spin-coated on the ETL substrates, then 150 μL of chlorobenzene with or without MOF@COF was poured on spinning substrate. Afterward, the films were annealed at 120 °C for different times (0, 3, 5, 10, 15, 20, 30, 60, 180, 300, 600, 1800, and 3600 s). The perovskite films annealed at different times were removed immediately for XRD test. The X-ray photoelectron spectroscopy (XPS) was performed with ESCLAB 250Xi from Thermo Fisher Scientific. The thermogravimetry (TGA) was carried out in a nitrogen or air stream using Netzsch thermal analysis equipment (STA449F5 Jupiter) with a heating rate of 5 °C $min^{-1}$. The different perovskite precursor solution with or without MOF@COF were heated at 100 °C for 1 h under $N_2$ condition to obtain in situ real-time TG-FTIR spectra. The density functional theory (DFT) was investigated by using the B3LYP functional and 6-31G (d, p) basis set (Gaussian 09 software). Scan electron microscope (SEM) measurements were carried out using Merlin Compact from ZEISS. The transmission electron microscopy (TEM) was performed using a FEI Tecnai G2 F30. The perovskite films based on $(FAPbI_3)_{0.93}(MAPbBr_3)_{0.04}(CsPbI_3)_{0.03}$ with or without MOF@COF were deposited on a clean FTO and then scratched from FTO. The perovskite power was dispersed in IPA and then dropped on TEM grids for testing. The perovskite films with or without MOF@COF were deposited on clean silicon chip for testing femtosecond transient absorption spectroscopy (fs-TAS). The atomic force microscope (AFM), Kelvin probe force microscopy (KPFM). UV-vis absorption spectra were carried out with a Shimadu and SPECORD S600 spectrophotometer. The PL emission intensity spectra were recorded by an FLS 920 luminescence spectrometer. The time-of-flight secondary-ion mass spectrometry (TOF-SIMS) measurement was performed using a TOF-SIMS V instrument (ION TOF-SIMS 5).

***Trap density Measurements***: The hole transport behavior could be also tested by the space-charge-limited current (SCLC) measurement in dark with a specific structure of FTO/$TiO_2$/perovskite/PCBM/Ag. The trap density ($N_t$) is calculated by following Eq. (S1):

$$N_t = \frac{2V_{TFL}\varepsilon_r\varepsilon_0}{qL^2} \tag{S1}$$

where $\varepsilon_0$ is the vacuum permittivity, $\varepsilon$ is the relative dielectric constant of perovskite, $q$ is the electron charge, and $L$ is the thickness of the perovskite film.

***TRPL decay spectra***: TRPL decay spectra of perovskite film without and with MOFs can be fitted with the following bi-exponential decay Eq. (S2):

$$y(t) = A_1 e^{-t/\tau 1} + A_2 e^{-t/\tau 2} + y_0 \tag{S2}$$

where $A_1$ and $A_2$ are the relative amplitudes, $\tau_1$ is the fast decay component, related to non-radiative recombination, $\tau_2$ is the slow decay component, related to radiative recombination, and $y_0$ is a constant for the base-line offset. The relevant key parameters resulting from the fitted results are listed in Table S5. The average carrier lifetimes ($\tau_{ave}$) can be calculated by the following formula:

$$\tau_{average} = (\tau_1^2 A_1 + \tau_2^2 A_2)/(\tau_1 A_1 + \tau_2 A_2) \tag{S3}$$

***Sorption kinetic***: MOF@COF (40.0 mg) and a freshly prepared aqueous solution of lead(II) iodide (10.0 ppm, 100.0 mL) were mixed in a 200 mL round-bottom flask and stirred at room temperature. During the adsorption, the mixture was withdrawn and filtered at intervals through a 0.22 μm membrane filter for all samples and then quantified by ICP-OES to determine the remaining Pb(II) content.

The linear formation of pseudo-first-order kinetic model kinetic model is given as following Eqs. (S5, S6).

$$ln(q_e - q_t) = ln\, q_t - k_1 t \tag{S5}$$

$$\frac{t}{q_t} = \frac{1}{k_2 q_e^2} + \frac{t}{q_e} \tag{S6}$$

where $k_1$ ($min^{-1}$) and $k_2$ ($g{\cdot}mg^{-1}{\cdot}min^{-1}$) refer to the rate constants of pseudo-first-order and pseudo-second-order kinetic models, $q_e$ and $q_t$ are the amounts of adsorbed uranyl ions ($mg{\cdot}g^{-1}$) at equilibrium and given time "t", respectively.

***Sorption isotherms***: Lead(II) acetate aqueous solutions of various Pb(II) concentrations (10, 25, 50, 100, 250, 500, 750 and 1000 mg $L^{-1}$) were prepared, and used in the following adsorption procedure. MOF@COF (5.0 mg) was added to each centrifuge tube containing Pb(II) solution (5.0 mL) with different concentrations. The mixtures were stirred at room temperature for 18 h. Then the supernatant was filtered through a 0.22-μm membrane and analyzed using ICP-OES to determine the remaining Pb(II) content. Generally, the Langmuir model, represented by Eq. (S7), assumes that there is no interaction between the adsorbate molecules and the adsorption localizes in a monolayer. Whereas Freundlich model proposes an empirical model that is based on adsorption on heterogeneous surfaces and can be expressed as Eq. (S8).

$$q_e = \frac{q_m K_L C_e}{1 + K_L C_e} \tag{S7}$$

$$q_e = K_F C_e^{1/n} \tag{S8}$$

where $q_e$ is the amount adsorbed at equilibrium (mg $g^{-1}$) and $C_e$ is the equilibrium concentration (mg $L^{-1}$), $q_m$ is the maximum adsorption amount or the saturated amount ($mg{\cdot}g^{-1}$), $K_L$ is an equilibrium constant related to the binding strength (L $mg^{-1}$), n and $K_F$ are Freundlich constants which are indicators of the adsorption capacity and adsorption intensity, respectively.

## S2 Supplementary Figures and Tables

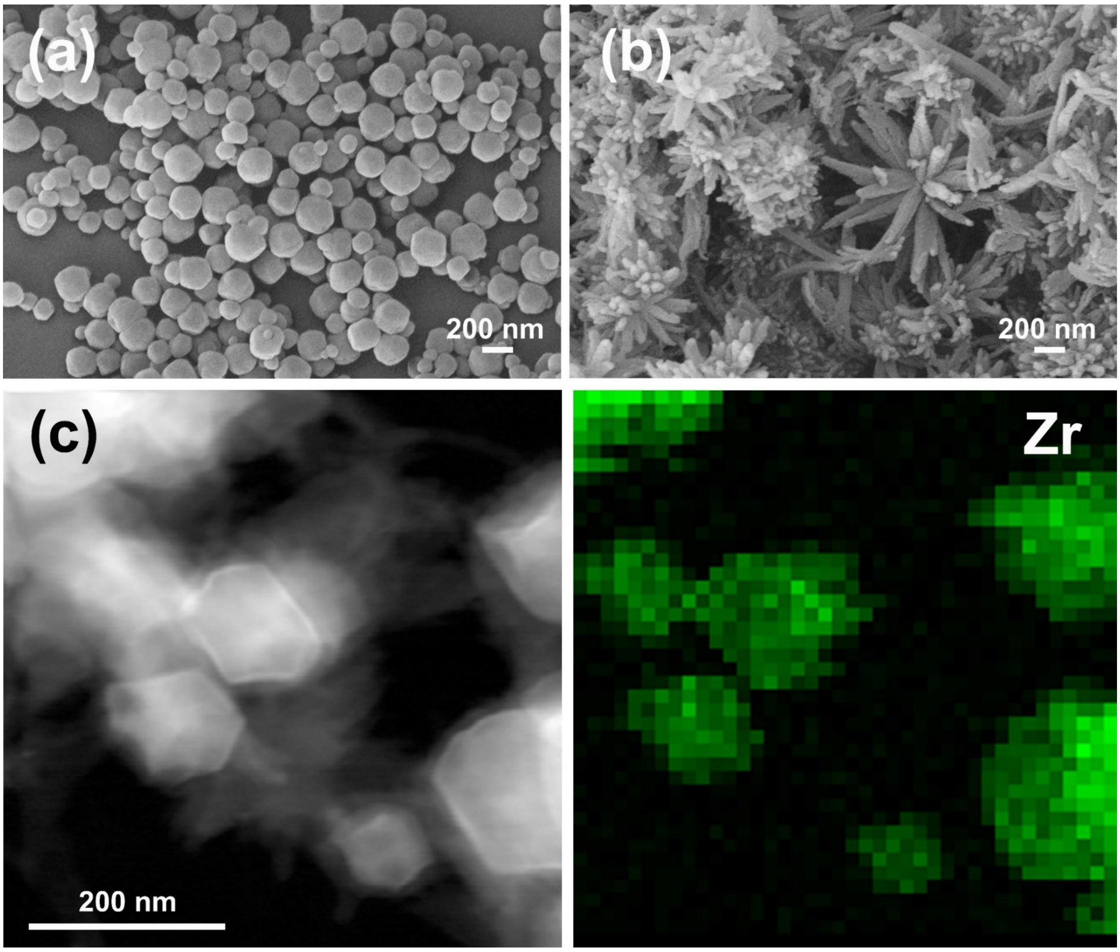


**Fig. S1** SEM images of (**a**) MOF-$NH_2$, (**b**) TpPa-1-COF, and (**c**) TEM mapping images of MOF@COF

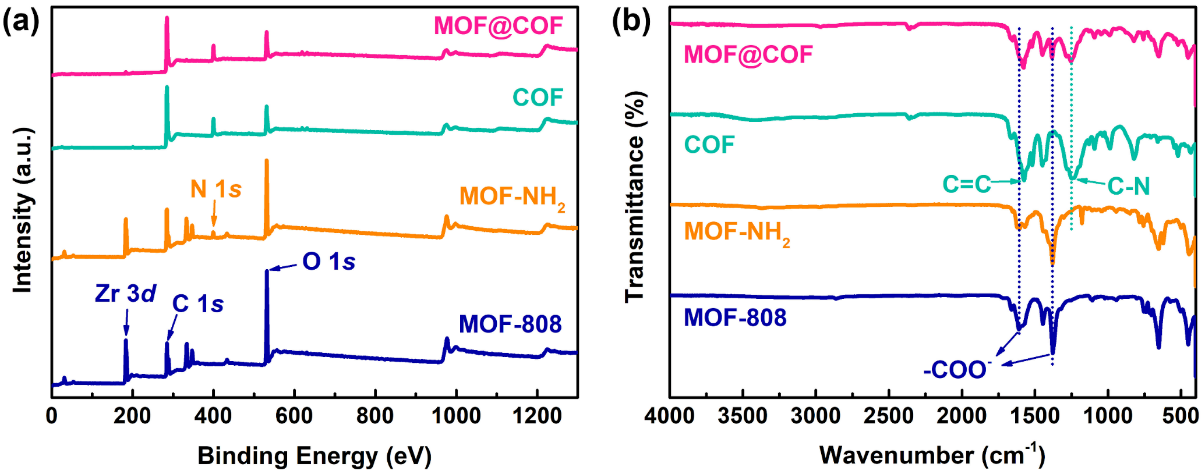


**Fig. S2** (**a**) XPS spectra and (**b**) IR spectra of MOF-808, $H_2H$-MOF-808, COF, and MOF@COF

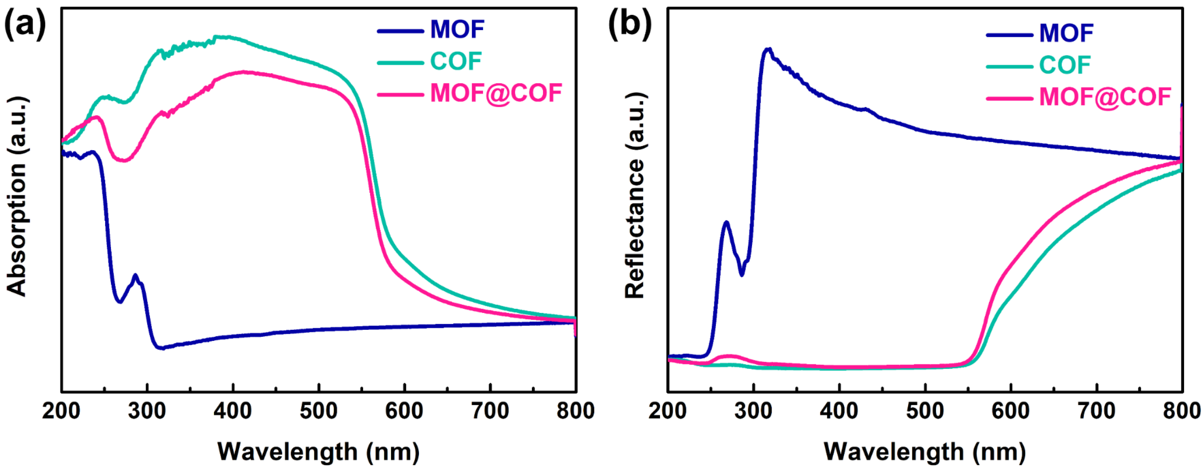


**Fig. S3** (**a**) UV-vis absorption spectra and (**b**) UV-vis diffuse reflectance spectra of MOF, COF and MOF@COF

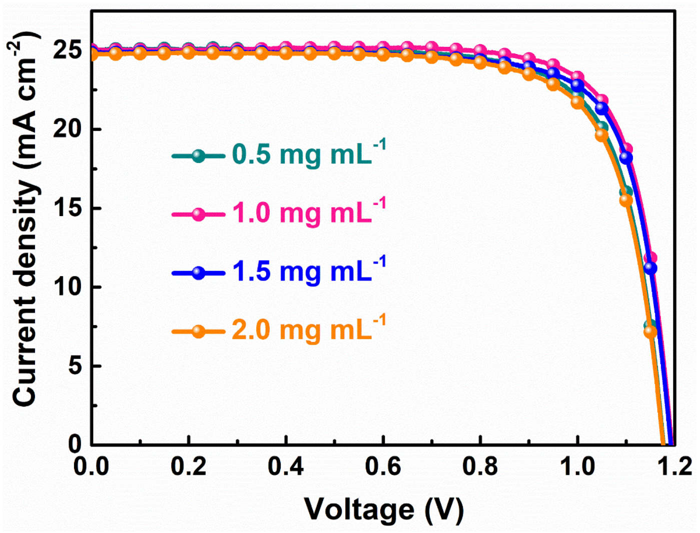


**Fig. S4** J-V curves of PSCs with different concentration of MOF@COF

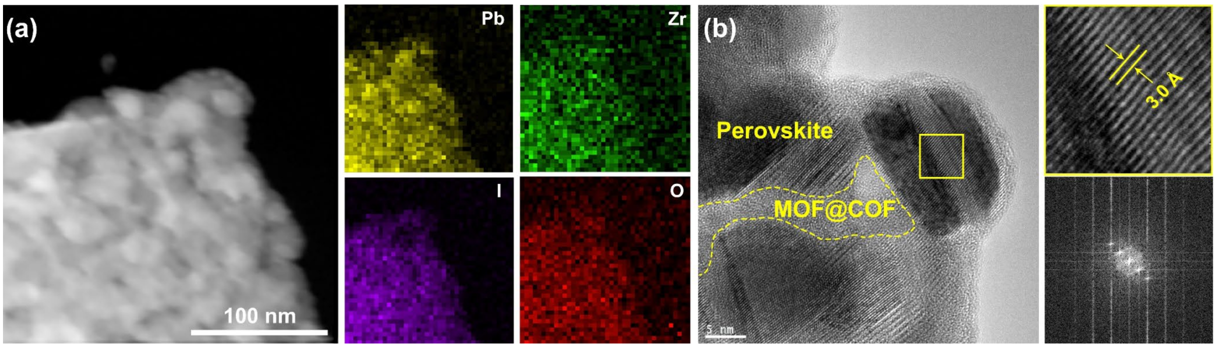


**Fig. S5** (**a**) TEM mapping and (**b**) HR-TEM images of MOF@COF functionalized perovskite film

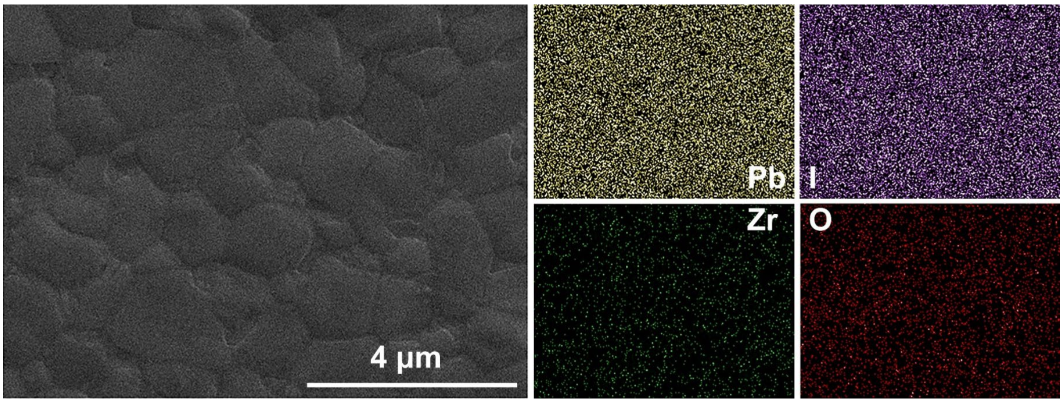


**Fig. S6** SEM mapping images of MOF@COF-functionalized perovskite film

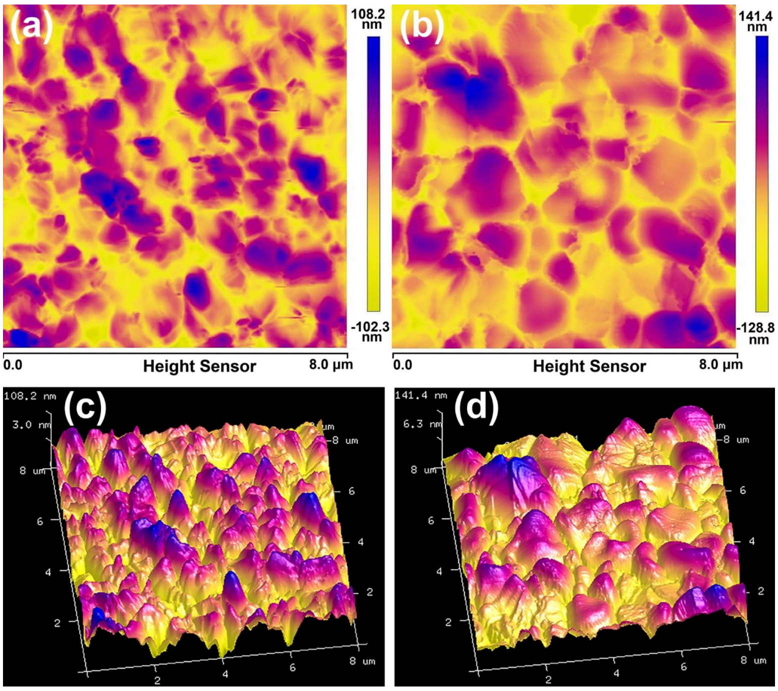


**Fig. S7** AFM images and 3D images of (**a, c**) control, (**b, d**) MOF@COF-functionalized perovskite films

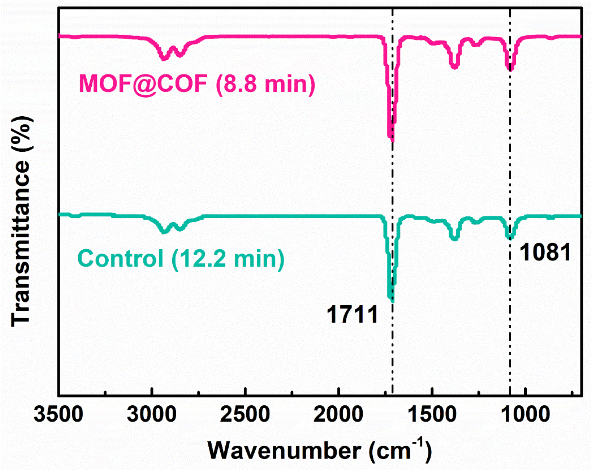


**Fig. S8** The selected FT-IR spectra of different perovskite precursors at maximum volatilization time

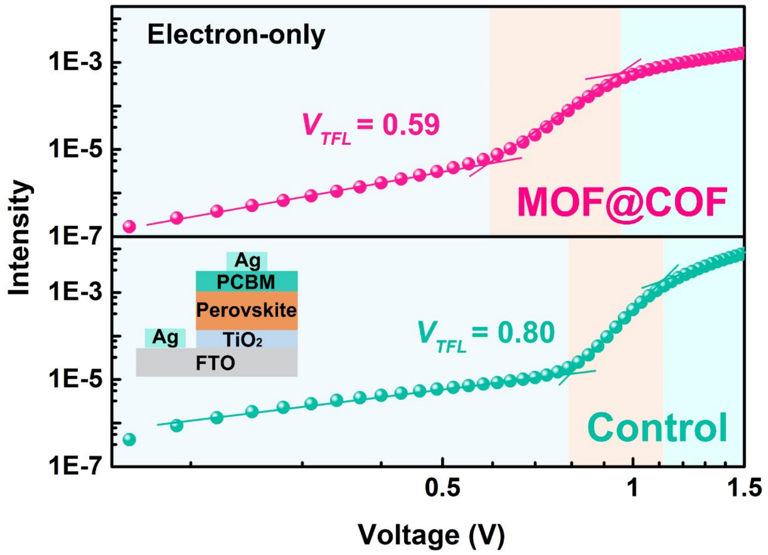


**Fig. S9** Dark J-V curves of the electron-only device

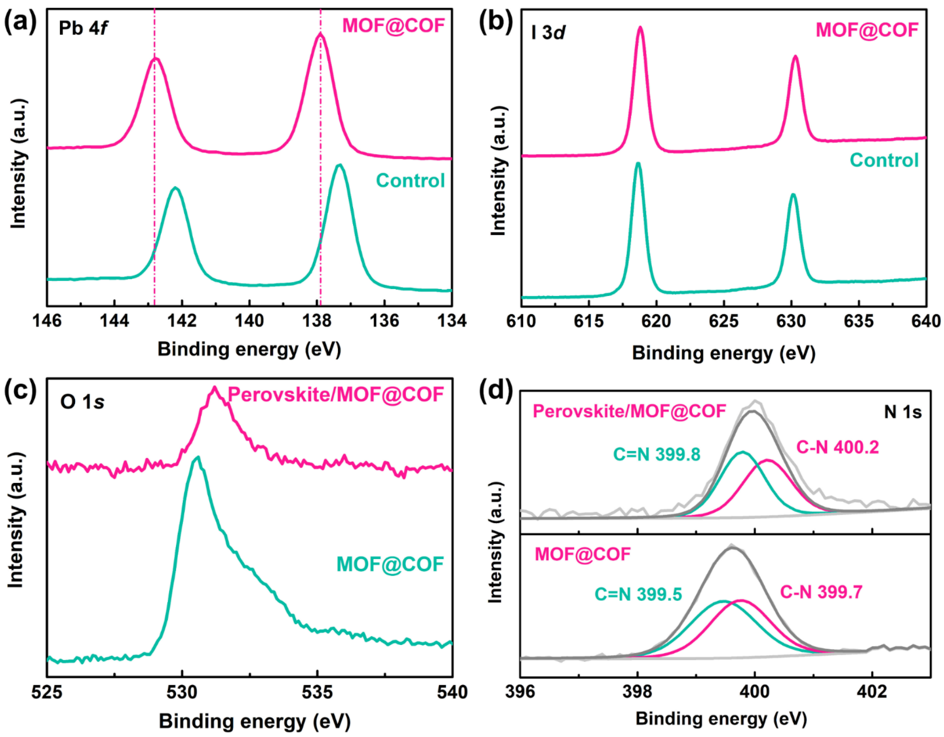


**Fig. S10** XPS spectra of (**a**) Pb 4f, (**b**) I 3d, (**c**) N 1s, and (**d**) N 1s of perovskite films without or with MOF@COF

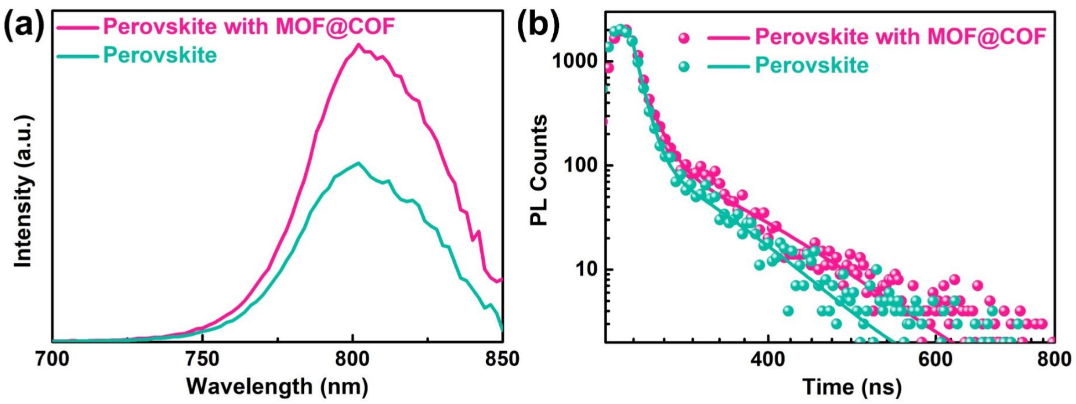


**Fig. S11** (**a**) PL and (**b**) TRPL spectra

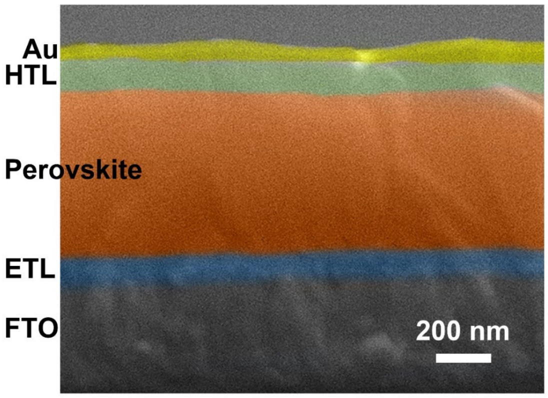


**Fig. S12** Cross-sectional SEM images of PSCs

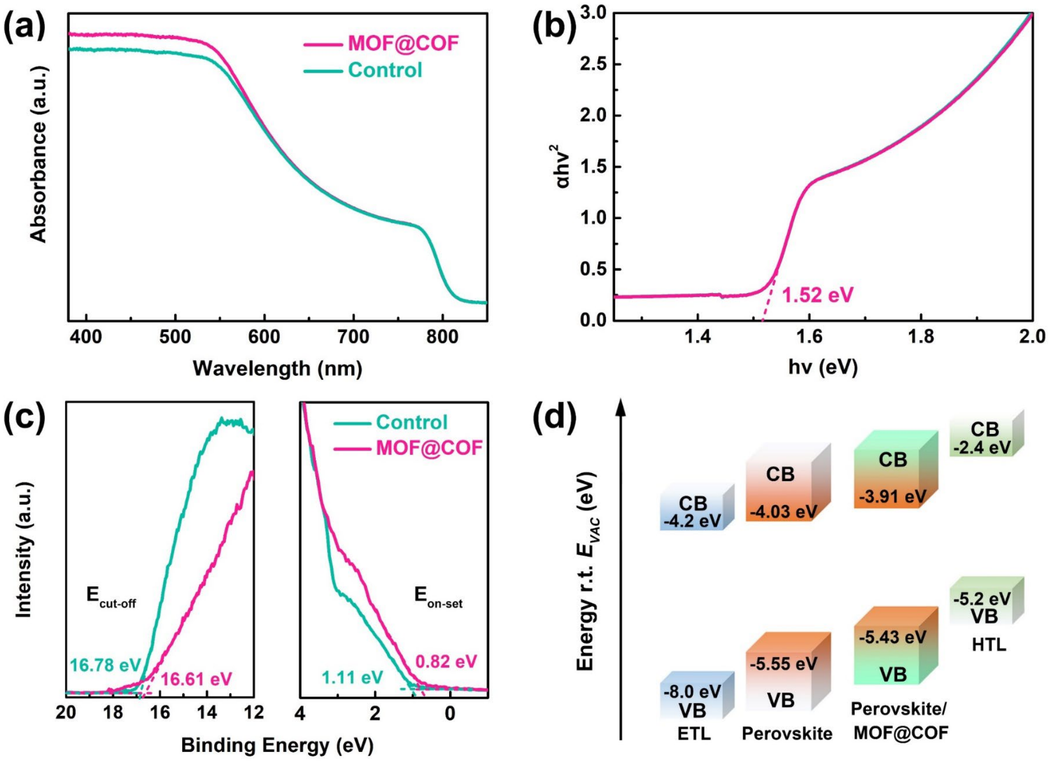


**Fig. S13** (**a**) UV-vis absorption spectra and (**b**) Tauc spectra of control and MOF@COF-functionalized perovskite films. (**c**) UPS spectra of control and MOF@COF functionalized

perovskite films. (**d**) Relevant energy level distribution of PSCs

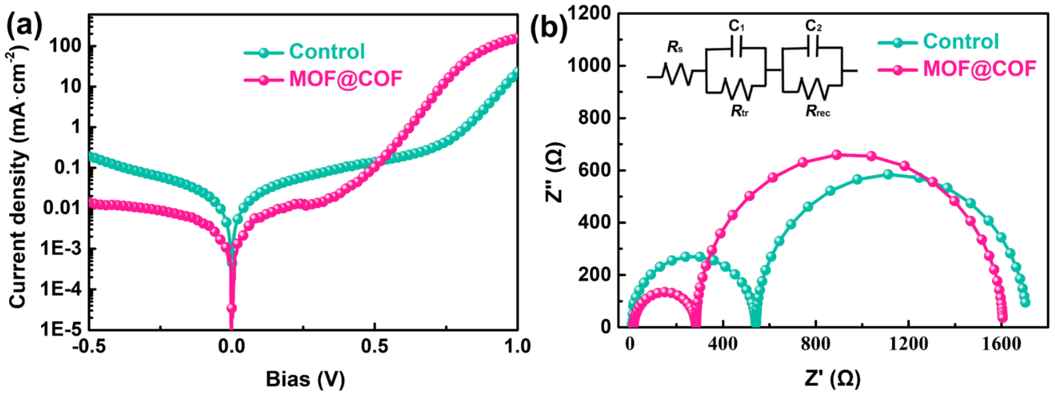


**Fig. S14** (**a**) J-V curves of PSCs with different concentration of MOF@COF under dark. (**b**) The EIS measurement spectra for control and MOF@COF-functionalized PSCs. (The Inset of Figure b is the equivalent electrical circuits for fitting the EIS data)

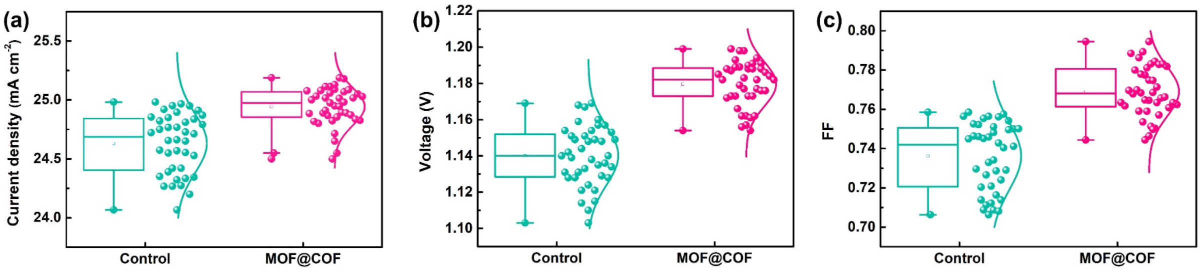


**Fig. S15** Statistical distribution of (**a**) $J_{SC}$, (**b**) $V_{OC}$, and (**c**) FF for control and MOF@COF-functionalized PSCs

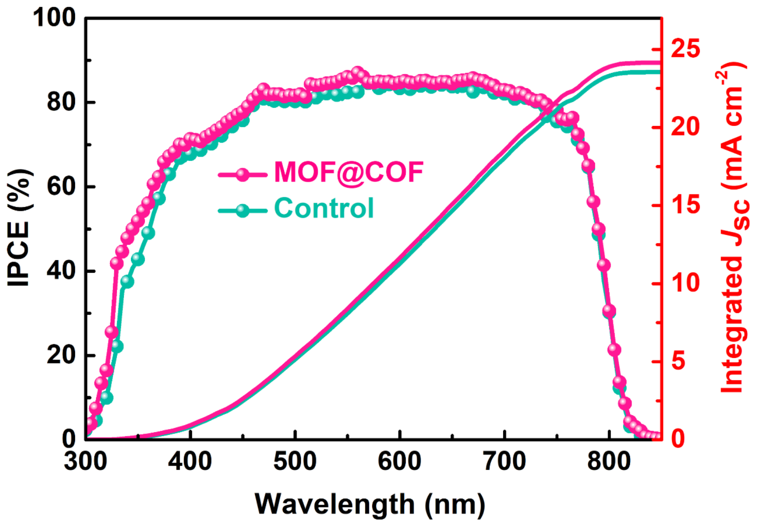


**Fig. S16** (**a**) IPCE curves and (**b**) integrated $J_{SC}$ of different PSCs

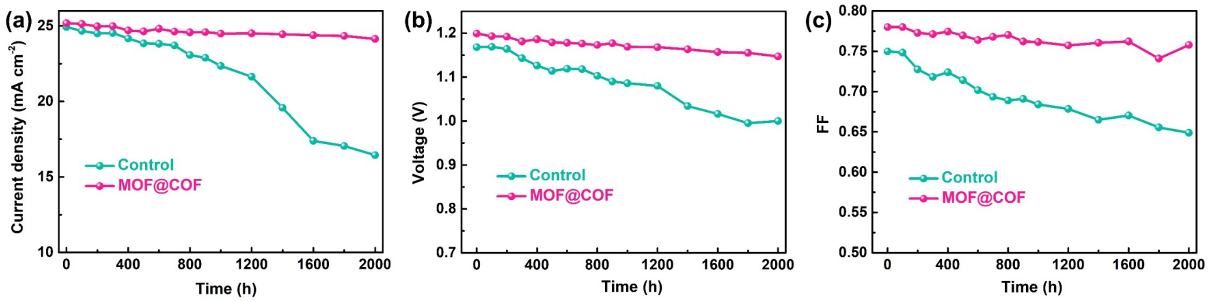


**Fig. S17** Long-term stability of control and MOF@COF-functionalized PSCs (stored conditions: 30%~50% RH and 25~30 °C). (**a**) $J_{SC}$, (**b**) $V_{OC}$, (**c**) FF

As we have proved the superior stabilities of the MOF@COF functionalized device under long-term storing conditions, we believe that the MOF@COF functionalized device would exhibit excellent stability under long-term operation. As is shown in Figure S18a, we have tracked the long-term stability test (MPP) of the unencapsulated devices with and without MOF@COF functionalization in ambient conditions under Am 1.5G simulated illumination for 200 h. It was found that the pristine device showed a rapid light decomposition within 200 h and only retained 51% of its PCE after the stability test. In comparison, the MOF@COF functionalized device maintained over 85% of its initial PCE under the same condition and only underwent a slight decrease of PCE during its working condition. As the performance loss of PSCs at MPP was frequently attributed to the cation and halide (vacancies) migration crossing the perovskite layer under the electric field, the results further suggested our efficient strategy of utilizing MOF@COF to enhance the perovskite quality with low-defect density, and evidently improve the operational stability of PSCs.

In a simulated thermal annealing at 85 °C, the MOF@COF functionalized unencapsulated devices possess superior stability compared to the pristine devices (Figure S18b). The pristine device maintained only 40% of its initial PCE after 300 h of aging. By contrast, the MOF@COF functionalized devices maintain over 80% of the initial efficiency after same time of aging, which is a substantial improvement compared to the pristine devices. The improvement in hydrothermal stability comes from the fact that the stability of MOF@COF could effectively restrain the penetration of water molecules into the perovskite layer, and enhanced the perovskite quality with low-defect density.

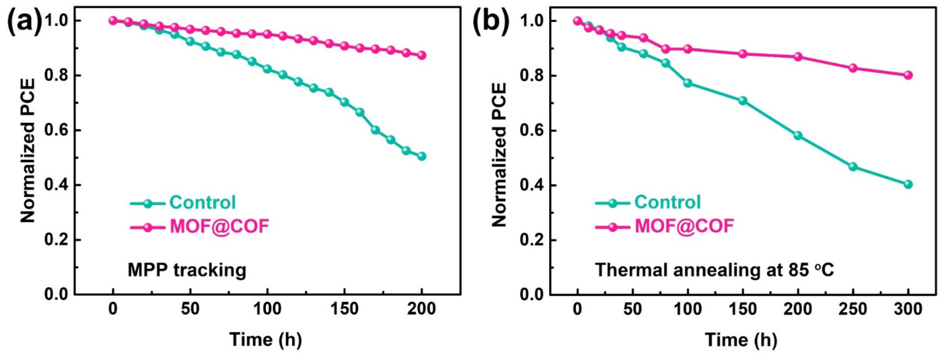


**Fig. S18** (**a**) The operational stability test of the un-encapsulated PSCs with or without MOF@COF under continuous illumination in the maximum power point (MPP) condition. (**b**) and thermal stability test the un-encapsulated PSCs with or without MOF@COF under thermal annealing at 85 °C

The $N_2$ adsorption and desorption experiments have been performed to evaluate the

permanent porosity of the synthetic materials (Fig. S19). The Brunauer–Emmett–Teller (BET) surface areas of MOF-808 and MOF@COF are 1037.7 and 753.5 $m^2$ $g^{-1}$, respectively. After introducing TpPa-1-COF, the reduced BET surface area is mainly ascribed to that TpPa-1-COF was in situ homogeneous growth on the surface of MOF-808. The high BET surface area of the resultant MOF@COF also shows the advantage of the combination of MOFs and COFs in retaining the porosity of the MOF/COF composite

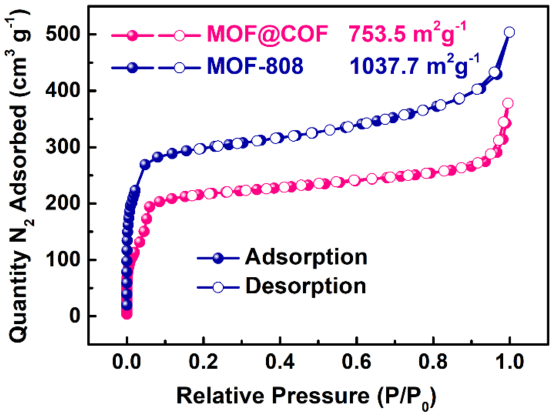


**Fig. S19** $N_2$ adsorption isotherms of MOF-808, and MOF@ COF

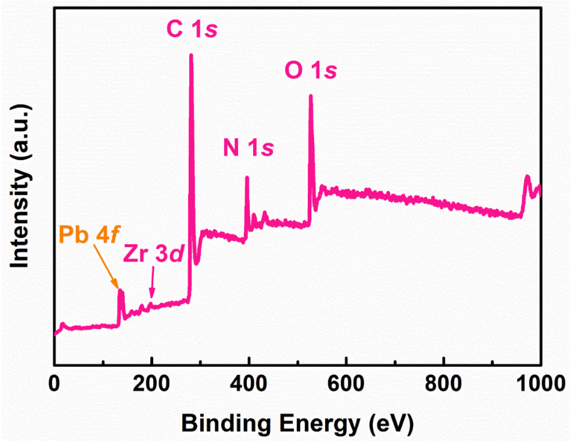


**Fig. S20** XPS spectrum of MOF@COF after adsorbing Pb

**Table S1** Photovoltaic parameters of control and MOF@COF-functionalized PSCs

| Dopant (mg·mL$^{-1}$) | $J_{sc}$ (mA·cm$^{-2}$) | $V_{oc}$ (V) | FF | PCE (%) |
|---|---|---|---|---|
| 0.5 | 25.02 | 1.15 | 0.78 | 22.67 |
| 1 | 25.18 | 1.20 | 0.78 | 23.61 |
| 1.5 | 25.08 | 1.18 | 0.75 | 22.15 |
| 2 | 24.80 | 1.18 | 0.74 | 21.81 |

**Table S2.** Carrier lifetime of control and MOF@COF-functionalized PSCs

| Devices | $\tau_1$ | $\tau_2$ | $A_1$% | $A_2$% | $\tau_{average}$[a] |
|---|---|---|---|---|---|
| Perovskite | 67 | 545 | 70.96 | 29.04 | 434 |
| Perovskite with MOF@COF | 106 | 748 | 36.79 | 63.21 | 699 |

[a] $\tau_{average} = (\tau_1^2A_1+\tau_2^2A_2)/(\tau_1A_1+\tau_2A_2)$

**Table S3** Summary of MOFs and POMs used in PSCs

| MOFs | | | | | |
|---|---|---|---|---|---|
| MOFs | $J_{sc}$ (mA·cm$^{-2}$) | $V_{oc}$ (V) | FF | PCE (%) | References |
| $Cu_3(HHTT)_2$ | 30.13 | 0.92 | 0.794 | 22.01 | [S3] |
| Cd-2D MOFs | 25.36 | 1.111 | 0.79 | 22.22 | [S4] |
| Zr-MOF-525/Perovsktie | 23.04 | 0.93 | 0.60 | 12.0 | [S5] |
| ZrL3 | 22.58 | 1.20 | 0.8138 | 22.02 | [S6] |
| Zn-CBOB | 23.17 | 1.135 | 0.784 | 20.64 | [S7] |
| mp-$TiO_2$/ZIF-8 | 22.82 | 1.02 | 0.73 | 16.99 | [S8] |
| Cu-BTC MOF | 21.8 | 1.09 | 0.75 | 17.8 | [S9] |
| UiO-66 | 21.85 | 1.072 | 0.769 | 18.01 | [S10] |
| Cu-BTC | 23.90 | 1.11 | 0.80 | 21.44 | [S11] |
| HKUST-1@mp-$TiO_2$ | 22.75 | 0.99 | 0.65 | 14.64 | [S12] |
| Eu-MOF | 23.71 | 1.14 | 0.82 | 22.16 | [S13] |
| $NH_2$-MIL-125 | 23.52 | 0.89 | 0.648 | 13.49 | [S14] |
| In-BTC | 23.55 | 1.12 | 0.79 | 20.87 | [S15] |
| ZnL | 23.86 | 1.118 | 0.793 | 21.15 | [S16] |
| Zn-TTB | 25.16 | 1.148 | 0.801 | 23.14 | [S17] |
| Pb-MOF | 24.37 | 1.11 | 0.762 | 20.87 | [S18] |
| PCN-224 QDs | 24.56 | 1.172 | 0.782 | 22.51 | [S19] |
| 2D-Zn-cbpp | 23.32 | 1.155 | 0.79 | 21.28 | [S20] |
| CoW12@MIL-101 | 23.85 | 1.135 | 0.79 | 21.39 | [S21] |
| MIL-125(Ti) | 22.81 | 1.01 | 0.718 | 16.56 | [S22] |
| ZIF-8@FAI | 23.93 | 1.058 | 0.756 | 19.13 | [S23] |
| Cd-Httb-BDC | 24.25 | 1.182 | 0.774 | 22.18 | [S24] |
| ZIF-8 | 22.13 | 1.06 | 0.72 | 17.32 | [S25] |
| POMOF | 25.03 | 1.194 | 0.78 | 23.30 | [S26] |
| 2D COF | 24.92 | 1.112 | 79.6 | 22.04 | [S27] |
| TTDA-TTA-COF | 24.64 | 1.14 | 79.95 | 22.44 | [S28] |
| SP-3D COF | 23.60 | 1.031 | 78.3 | 19.07 | [S29] |
| Car-ETTA | 23.18 | 1.097 | 77.84 | 19.79 | [S30] |
| DA-COF-2 | 24.55 | 1.10 | 81.96 | 21.11 | [S31] |
| HS-COFs | 25.10 | 1.17 | 82.6 | 24.26 | [S32] |
| COF | 14.17 | 1.282 | 79.01 | 14.35 | [S33] |
| MOF@COF | 25.18 | 1.20 | 78.0 | 23.61 | This work |

**Table S4** Photovoltaic parameters of control and MOF@COF-functionalized PSCs in the forward and reverse scanning directions

| Device | Scan directions | $J_{sc}$ (mA·cm$^{-2}$) | $V_{oc}$ (V) | FF | PCE (%) | Hysteresis index[a] |
|---|---|---|---|---|---|---|
| Control | Forward scan | 24.10 | 1.11 | 0.71 | 18.97 | 0.129 |
| | Reverse scan | 24.92 | 1.17 | 0.75 | 21.78 | |
| MOF@ COF | Forward scan | 24.88 | 1.18 | 0.76 | 22.24 | 0.058 |
| | Reverse scan | 25.18 | 1.20 | 0.78 | 23.61 | |

[a] Hysteresis index = $(PCE_{Reverse} - PCE_{Forward})/PCE_{Reverse}$.

## Supplementary References

[S1] J.H. Chong, M. Sauer, B.O. Patrick, M.J. MacLachlan, Highly stable keto-enamine salicylideneanilines. Org. Lett. **5**, 3823–3826 (2003). https://doi.org/10.1021/ol0352714

[S2] H.-Y. Zhang, Y. Yang, C.-C. Li, H.-L. Tang, F.-M. Zhang et al., A new strategy for constructing covalently connected MOF@COF core–shell heterostructures for enhanced photocatalytic hydrogen evolution. J. Mater. Chem. A **9**, 16743–16750 (2021). https://doi.org/10.1039/d1ta04493a

[S3] J. Cao, C.-K. Liu, V. Piradi, H.-L. Loi, T. Wang et al., Ultrathin self-assembly two-dimensional metal–organic framework films as hole transport layers in ideal-bandgap perovskite solar cells. ACS Energy Lett. **7**, 3362–3369 (2022). https://doi.org/10.1021/acsenergylett.2c01714

[S4] J. Ji, B. Liu, H. Huang, X. Wang, L. Yan et al., Nondestructive passivation of the $TiO_2$ electron transport layer in perovskite solar cells by the PEIE-2D MOF interfacial modified layer. J. Mater. Chem. C **9**, 7057–7064 (2021). https://doi.org/10.1039/d1tc00036e

[S5] T.-H. Chang, C.-W. Kung, H.-W. Chen, T.-Y. Huang, S.-Y. Kao et al., Planar heterojunction perovskite solar cells incorporating metal–organic framework nanocrystals. Adv. Mater. **27**, 7229–7235 (2015). https://doi.org/10.1002/adma.201502537

[S6] S. Wu, Z. Li, M.-Q. Li, Y. Diao, F. Lin et al., 2D metal–organic framework for stable perovskite solar cells with minimized lead leakage. Nat. Nanotechnol. **15**, 934–940 (2020). https://doi.org/10.1038/s41565-020-0765-7

[S7] J. Wang, J. Zhang, Y. Yang, S. Gai, Y. Dong et al., New insight into the lewis basic sites in metal–organic framework-doped hole transport materials for efficient and stable perovskite solar cells. ACS Appl. Mater. Interfaces **13**, 5235–5244 (2021). https://doi.org/10.1021/acsami.0c19968

[S8] D. Shen, A. Pang, Y. Li, J. Dou, M. Wei Metal–organic frameworks at interfaces of hybrid perovskite solar cells for enhanced photovoltaic properties. Chem. Commun. **54**, 1253–1256 (2018). https://doi.org/10.1039/c7cc09452c

[S9] J. Lee, N. Tsvetkov, S.R. Shin, J.K. Kang Fast charge transfer and high stability via hybridization of hygroscopic Cu-BTC metal–organic framework nanocrystals with a light-absorbing layer for perovskite solar cells. ACS Appl. Mater. Interfaces **14**, 35495–35503 (2022). https://doi.org/10.1021/acsami.2c05488

[S10] C.-C. Lee, C.-I. Chen, Y.-T. Liao, K.C.-W. Wu, C.-C. Chueh Perovskite solar cells: enhancing efficiency and stability of photovoltaic cells by using perovskite/Zr-MOF heterojunction including bilayer and hybrid structures. Adv. Sci. **6**, 1970030 (2019). https://doi.org/10.1002/advs.201970030

[S11] Y. Dong, J. Zhang, Y. Yang, L. Qiu, D. Xia et al., Self-assembly of hybrid oxidant POM@Cu-BTC for enhanced efficiency and long-term stability of perovskite solar cells. Angew. Chem. Int. Ed. **58**, 17610–17615 (2019). https://doi.org/10.1002/anie.201909291

[S12] H. Amrollahi Bioki, A. Moshaii, M. Borhani Zarandi, Performance improvement of ambient-condition fabricated perovskite solar cells using an interfacial HKUST-1 MOF on electron transfer layer. Surf. Interfaces **27**, 101579 (2021). https://doi.org/10.1016/j.surfin.2021.101579

[S13] J. Dou, C. Zhu, H. Wang, Y. Han, S. Ma et al., Synergistic effects of Eu-MOF on perovskite solar cells with improved stability. Adv. Mater. **33**, 2102947 (2021). https://doi.org/10.1002/adma.202102947

[S14] B. Li, J. Zhao, Q. Lu, S. Zhou, H. Wei et al., Carbon-based printable perovskite solar cells with a mesoporous $TiO_2$ electron transporting layer derived from metal–organic framework $NH_2$-MIL-125. Energy Technol. **9**, 2000957 (2021). https://doi.org/10.1002/ente.202000957

[S15] N.R. Chodankar, S.-H. Ji, Y.-K. Han, D.-H. Kim, Dendritic nanostructured waste copper wires for high-energy alkaline battery. Nano-Micro Lett. **12**, 1 (2019). https://doi.org/10.1007/s40820-019-0337-2

[S16] C. Li, J. Qiu, M. Zhu, Z. Cheng, J. Zhang et al., Multifunctional anionic metal-organic frameworks enhancing stability of perovskite solar cells. Chem. Eng. J. **433**, 133587 (2022). https://doi.org/10.1016/j.cej.2021.133587

[S17] J. Wang, J. Zhang, S. Gai, W. Wang, Y. Dong et al., Self-organized small molecules in robust MOFs for high-performance perovskite solar cells with enhanced degradation activation energy. Adv. Funct. Mater. **32**, 2203898 (2022). https://doi.org/10.1002/adfm.202203898

[S18] C.-K. Liu, K.-H. Wu, Y.-A. Lu, L.-Y. Hsiao, K.-W. Lai et al., Introducing postmetalation metal–organic framework to control perovskite crystal growth for efficient perovskite solar cells. ACS Appl. Mater. Interfaces **13**, 60125–60134 (2021). https://doi.org/10.1021/acsami.1c22144

[S19] Y. Liu, T. Liu, X. Guo, M. Hou, Y. Yuan et al., Porphyrinic metal–organic framework quantum dots for stable n–i–p perovskite solar cells. Adv. Funct. Mater. **33**, 2210028 (2023). https://doi.org/10.1002/adfm.202210028

[S20] L. Qiu, K. Xing, J. Zhang, Y. Yang, W. Cao et al., Two-dimensional metal–organic frameworks-based grain termination strategy enables high-efficiency perovskite photovoltaics with enhanced moisture and thermal stability. Adv. Funct. Mater. **31**, 2010368 (2021). https://doi.org/10.1002/adfm.202010368

[S21] W. Wang, J. Zhang, K. Lin, Y. Dong, J. Wang et al., Construction of polyoxometalate-based material for eliminating multiple Pb-based defects and enhancing thermal stability of perovskite solar cells. Adv. Funct. Mater. **31**, 2105884 (2021). https://doi.org/10.1002/adfm.202105884

[S22] X. Hou, L. Pan, S. Huang, O.-Y. Wei, X. Chen, Enhanced Efficiency and stability of Perovskite Solar Cells using Porous Hierarchical $TiO_2$ Nanostructures of Scattered

Distribution as Scaffold. Electrochim. Acta **236**, 351–358 (2017). https://doi.org/10.1016/j.electacta.2017.03.192

[S23] C. Li, S. Guo, J. Chen, Z. Cheng, M. Zhu et al., Mitigation of vacancy with ammonium salt-trapped ZIF-8 capsules for stable perovskite solar cells through simultaneous compensation and loss inhibition. Nanoscale Adv. **3**, 3554–3562 (2021). https://doi.org/10.1039/d1na00173f

[S24] Y. Dong, S. Gai, J. Zhang, R. Fan, B. Hu et al., Metal-organic frameworks with mixed-ligands strategy as heterogeneous nucleation center to assist crystallization for efficient and stable perovskite solar cells. J. Energy Chem. **77**, 1–10 (2023). https://doi.org/10.1016/j.jechem.2022.10.029

[S25] Z. Zhang, X. Luo, B. Wang, J. Zhang, Electron transport improvement of perovskite solar cells via a ZIF-8-derived porous carbon skeleton. ACS Appl. Energy Mater. **2**, 2760–2768 (2019). https://doi.org/10.1021/acsaem.9b00098

[S26] Y. Dong, J. Zhang, W. Wang, B. Hu, D. Xia et al., Regulating crystallization and lead leakage of perovskite solar cell via novel polyoxometalate-based metal–organic framework. Small **19**, 2301824 (2023). https://doi.org/10.1002/smll.202301824

[S27] J. He, H. Liu, F. Zhang, X. Li, S. Wang, *In situ* synthesized 2D covalent organic framework nanosheets induce growth of high-quality perovskite film for efficient and stable solar cells. Adv. Funct. Mater. **32**, 2110030 (2022). https://doi.org/10.1002/adfm.202110030

[S28] R. Nie, W. Chu, Z. Li, H. Li, S. Chen et al., Simultaneously suppressing charge recombination and decomposition of perovskite solar cells by conjugated covalent organic frameworks. Adv. Energy Mater. **12**, 2200480 (2022). https://doi.org/10.1002/aenm.202200480

[S29] C. Wu, Y. Liu, H. Liu, C. Duan, Q. Pan et al., Highly conjugated three-dimensional covalent organic frameworks based on spirobifluorene for perovskite solar cell enhancement. J. Am. Chem. Soc. **140**, 10016–10024 (2018). https://doi.org/10.1021/jacs.8b06291

[S30] M.G. Mohamed, C.-C. Lee, A.F.M. EL-Mahdy, J. Lüder, M.-H. Yu et al., Exploitation of two-dimensional conjugated covalent organic frameworks based on tetraphenylethylene with bicarbazole and pyrene units and applications in perovskite solar cells. J. Mater. Chem. A **8**, 11448–11459 (2020). https://doi.org/10.1039/d0ta02956d

[S31] Z. Li, Z. Zhang, R. Nie, C. Li, Q. Sun et al., Construction of stable donor–acceptor type covalent organic frameworks as functional platform for effective perovskite solar cell enhancement. Adv. Funct. Mater. **32**, 2112553 (2022). https://doi.org/10.1002/adfm.202112553

[S32] J. Guo, G. Meng, X. Zhang, H. Huang, J. Shi et al., Dual-interface modulation with covalent organic framework enables efficient and durable perovskite solar cells. Adv. Mater. **35**, 2302839 (2023). https://doi.org/10.1002/adma.202302839

[S33] J. Zhang, J. Duan, Q. Guo, Q. Zhang, Y. Zhao et al., A universal grain “cage” to suppress halide segregation of mixed-halide inorganic perovskite solar cells. ACS Energy Lett. **7**, 3467–3475 (2022). https://doi.org/10.1021/acsenergylett.2c01771
